# A scientometrics analysis and visualization of refractory gastroesophageal reflux disease

**DOI:** 10.3389/fphar.2024.1393526

**Published:** 2024-07-30

**Authors:** Nan Zhang, Ming Han, Qin-Wei Zheng, Meng-Yuan Zhang, Wen-Lan Zhi, Jing-Jing Li, Lin-Xuan Cui, Jin-Li Tian, Yi Wang, Sheng-Quan Fang

**Affiliations:** Department of Gastroenterology, Yueyang Hospital of Integrated Traditional Chinese and Western Medicine, Shanghai University of Traditional Chinese Medicine, Shanghai, China

**Keywords:** refractory GERD, scientometrics analysis, visualization, Citespace, research hotspots

## Abstract

**Background:**

Refractory gastroesophageal reflux disease (refractory GERD) is a heterogeneous disease characterized by unresponsiveness or poor efficacy to proton-pump inhibitors (PPIs). This chronic disorder substantially weakens patients’ mental wellbeing and quality of life, increasing the financial burden on society. Multiple articles have been reported in this area. However, literature involving scientometric analysis of refractory GERD is absent. Therefore, it is necessary to understand the evolution of research themes and the main hotspots of refractory GERD through bibliometric methods.

**Methods:**

All documents related to refractory GERD based on the WOS Core Collection from January 2000 to November 2023 were selected for analysis. Citespace V 6.1 R6, VOSviewer V 1.6.20, and Scimago Graphica V 1.0.38 were used to perform bibliometric analysis.

**Results:**

We collected a total of 241 research articles from 36 countries and 322 institutions, contributed by over 1,000 authors. Over the last 20 years, the number of articles in this field has increased year by year, and since 2011, the number of publications has increased dramatically, with 85.89% of the papers. These countries are led by the United States and Japan. *GUT* had the highest number of citations and *DIGESTION* had the highest number of publications. Research on standardized diagnosis and management, mechanisms, novel monitoring methods, and innovative drugs and procedures for refractory GERD are the main topics and hotspots in this field. This study also found that neuroimmune interaction is closely related to refractory GERD, which may be a new direction for future mechanism research.

**Conclusion:**

Our study is the first bibliometric analysis of the global literature on refractory GERD. This research provides valuable insights for researchers, enabling them to quickly understand the research frontier and hot topics of this field.

## 1 Introduction

Refractory gastroesophageal reflux disease (refractory GERD) is a heterogeneous disease characterized by unresponsiveness or poor efficacy to proton-pump inhibitors (PPIs). The latest update of the Lyon Consensus 2.0 identifies heartburn, oesophageal chest pain, and regurgitation as typical symptoms, and atypical manifestations such as belching and chronic cough also exhibit a potential pathophysiological association with this condition (Gyawali et al., 2023). As a distinct subtype of gastroesophageal reflux disease, the persistence of symptoms and the variability in treatment efficacy pose challenges for clinicians in daily management and therapy (Armstrong et al., 2022). Consequently, the diagnosis and treatment of refractory patients have become prominent areas of challenge and difficulty within the field of gastrointestinal diseases.

One study reported that the global prevalence of GERD in 2020 was about 13.98% (Nirwan et al., 2020). According to the World Population Prospects issued by the United Nations in 2022, it is estimated that over 1 billion individuals will be affected by GERD, with approximately 13.2%–54.1% of them being non-responsive or inadequately responsive to long-term PPIs treatment (Chey et al., 2010; Katz et al., 2013a; Kahrilas et al., 2013; Delshad et al., 2020). In the United States and the United Kingdom, patients with refractory GERD have significantly more visits to primary care facilities and emergency departments, significantly reducing patient productivity and sleep quality (Toghanian et al., 2011; Kahrilas et al., 2013; Wang HM. et al., 2023). Compared to patients who responded positively to PPIs therapy, those with refractory responses incurred an average of $7,000 to $10,000 higher in associated healthcare costs and were also more susceptible to gastrointestinal bleeding, dysphagia, and other related conditions (Gerson et al., 2011; Howden et al., 2021). In addition, long-term PPIs use as well as referrals for further treatment not only directly impose financial burdens on patients but also exert a considerable negative impact on their mental health and overall quality of life while simultaneously imposing additional financial strains on society.

With the growing attention in this field, researchers have made more in-depth explorations in this field every year to have a more thorough understanding of this disease. Simultaneously, numerous related articles have been published, posing a challenge on how to efficiently comprehend research topics and identify potential directions in this field. Bibliometric methods offer unique advantages in addressing this issue effectively. However, no bibliometric analysis of refractory GERD has been reported so far.

Here, we employed bibliometric methods to analyze the research status and development history of refractory GERD from 2000 to 2023. Through manual classification and summarization, we demonstrate scientific collaboration among institutions and regions while identifying key research areas such as standardized diagnosis and management, novel mechanisms and detection methods, as well as the development of new drugs and surgical procedures. These findings highlight the focal points of research in this field along with future trends.

## 2 Data and methods

### 2.1 Data sources and search strategies

Web of Science (WOS) is a database and research tool with comprehensive literature content and strong influence, which can help us quickly locate high-impact papers, understand the latest progress in the field, and find breakthroughs in research ideas (Li et al., 2018). Therefore, the WOS Core Collection was used as the source of literature data in this study. The search time span was from January 2000 to November 2023, and the search strategy was as follows: TIm = (Refractory OR resistant OR “no respon*” OR “not respond” OR “Non-Responsive” OR Ineffective OR “No effect”) AND TI=(“Gastroesophageal reflux disease” OR GERD OR “Gastric esophageal reflux” OR GORD OR “Non*erosive Reflux Disease” OR NERD OR “Endoscopy-negative reflux” OR “Endoscopy normal reflux” OR Heartburn OR “Reflux Esophag*” OR “Erosive Reflux” OR “Erosive Esophag*” OR “Oesophageal reflux” OR “Barrett’s esophag*” OR “Gastric Acid Reflux” OR “Acid Reflux” OR Reflux OR “Proton Pump Inhibitor” OR PPI). The search time was 10 December 2023, and a total of 842 records were retrieved.

### 2.2 Data extraction and collection

**FIGURE 1:**
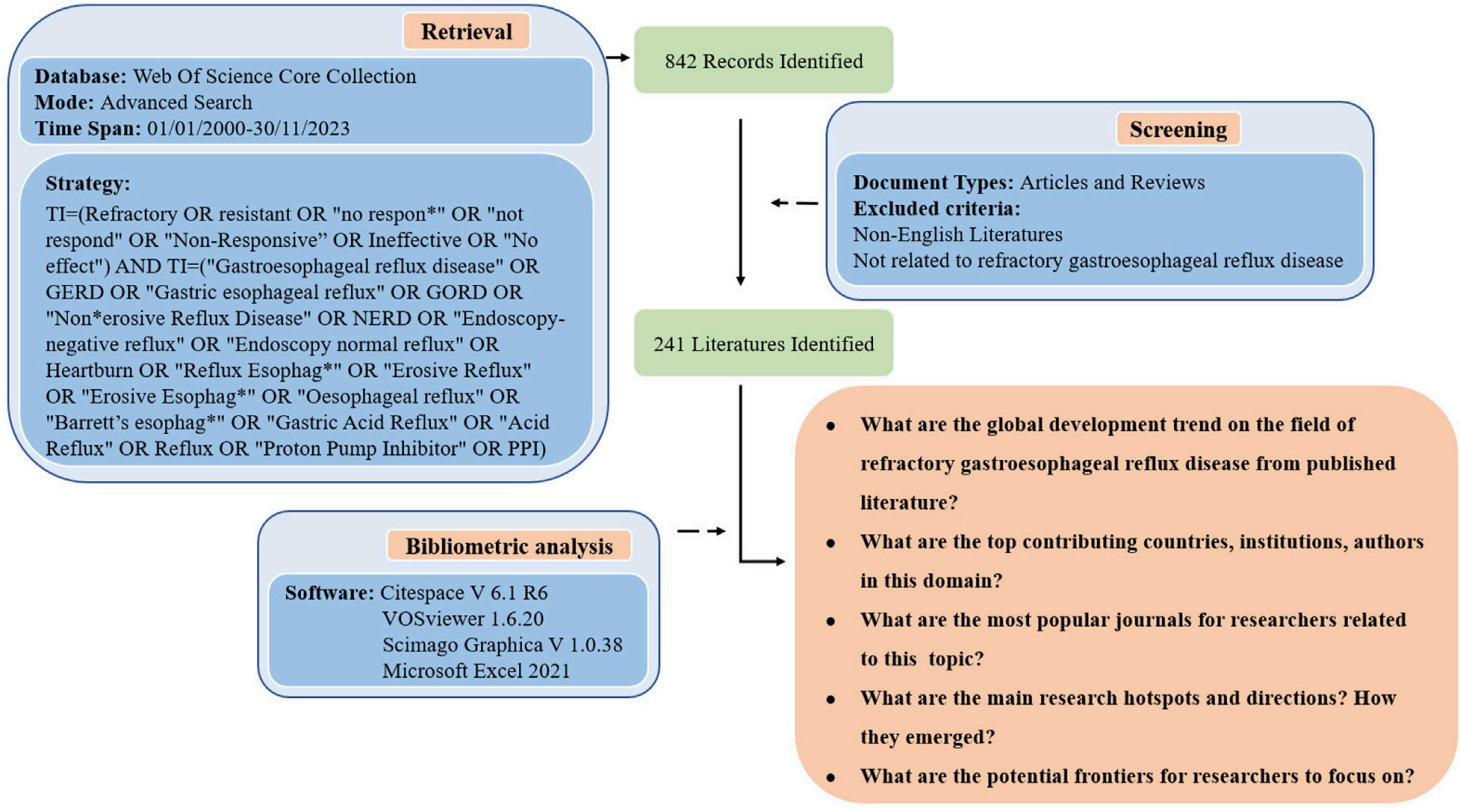
Flowchart of the literature-screening process and research framework.

The following document types were excluded from this study: Proceeding Paper or Meeting Abstract or Letter or Book Chapters or Early Access or Editorial Material or Reprint or Meeting Summary, only articles and reviews in English were retained. Although TI can accurately search the literature, there are still some articles that are not related to refractory GERD. After screening and checking by two researchers, the literature whose content did not conform to the research theme was excluded. A total of 601 articles were excluded, and 241 publications were included for bibliometric analysis. Figure 1 shows the flowchart of the literature-screening process and research framework.

### 2.3 Bibliometric analysis

The data were imported into the corresponding analysis software for bibliometric and visualization analysis. The software includes Citespace V 6.1 R6, VOSviewer V 1.6.20, and Scimago Graphica V 1.0.38. These tools excel at presenting the intricate and extensive development of a specific field through visualization techniques, enabling quick identification of key information and pivotal moments to predict scientific hotspots and frontiers. In this study, Citespace V 6.1R6 made the keyword cluster map, Timeline view of keywords map, and analyzed the burst citations. VOSviewer 1.6.20 was used to analyze institutions, collaborative relationships, and co-cited references. Scimago Graphica V 1.0.38 shows the network of cooperation between countries. Microsoft Excel 2021 was used for the top 10 cited references, the top 10 cited authors, and the top 15 journals by number of publications. Complementary information and visual images were integrated across different software platforms to comprehensively and scientifically analyze the literature data. The data underwent preprocessing before conducting analysis. Nonsensical keywords (e.g., “disease,” “health,” “esophagus”) were excluded. Keywords with similar concepts but varying expressions or spellings were combined for analysis purposes (e.g., “gastroesophageal reflux disease,” “GERD,” “GORD”; “pH/mii,” “impedance-pH monitoring”).

## 3 Results

### 3.1 Annual publication and citation trends

**FIGURE 2:**
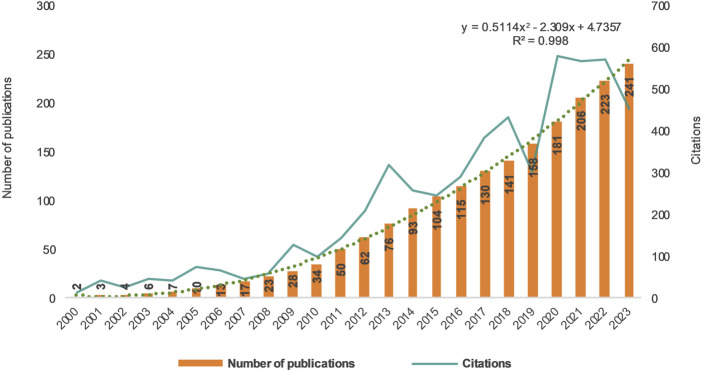
Trends in the growth of the publications and numbers of cited articles worldwide from 2000 to 2023.

This study examined articles on refractory GERD for 24 years, from 2000 to 2023. In the initial 11-year period, only 14.11% of all articles were published, whereas in the subsequent 13 years from 2011 until 2023, the number of articles accounted for 85.89%. Figure 2 illustrates that a turning point occurred in this field in 2011 when the number of publications rapidly increased from 6 to 17. The quantity of publications has reached a new peak, rising from 2 in 2000 to 24 in 2021. Since 2019, there has been consistent annual publication exceeding 17 articles and reaching its highest citation count at an impressive figure of 581. The percentage of review articles and articles was 11.62% and 88.38% respectively. By employing curve regression modeling, this study generated a polynomial function growth curve representing the yearly expansion of literature and found it to be highly consistent with the actual growth trend observed within these publications (R^2^ = 0.998), thus confirming the annual progression pattern concerning articles and research on refractory GERD.

### 3.2 Analysis of countries or regions and institutions

**FIGURE 3:**
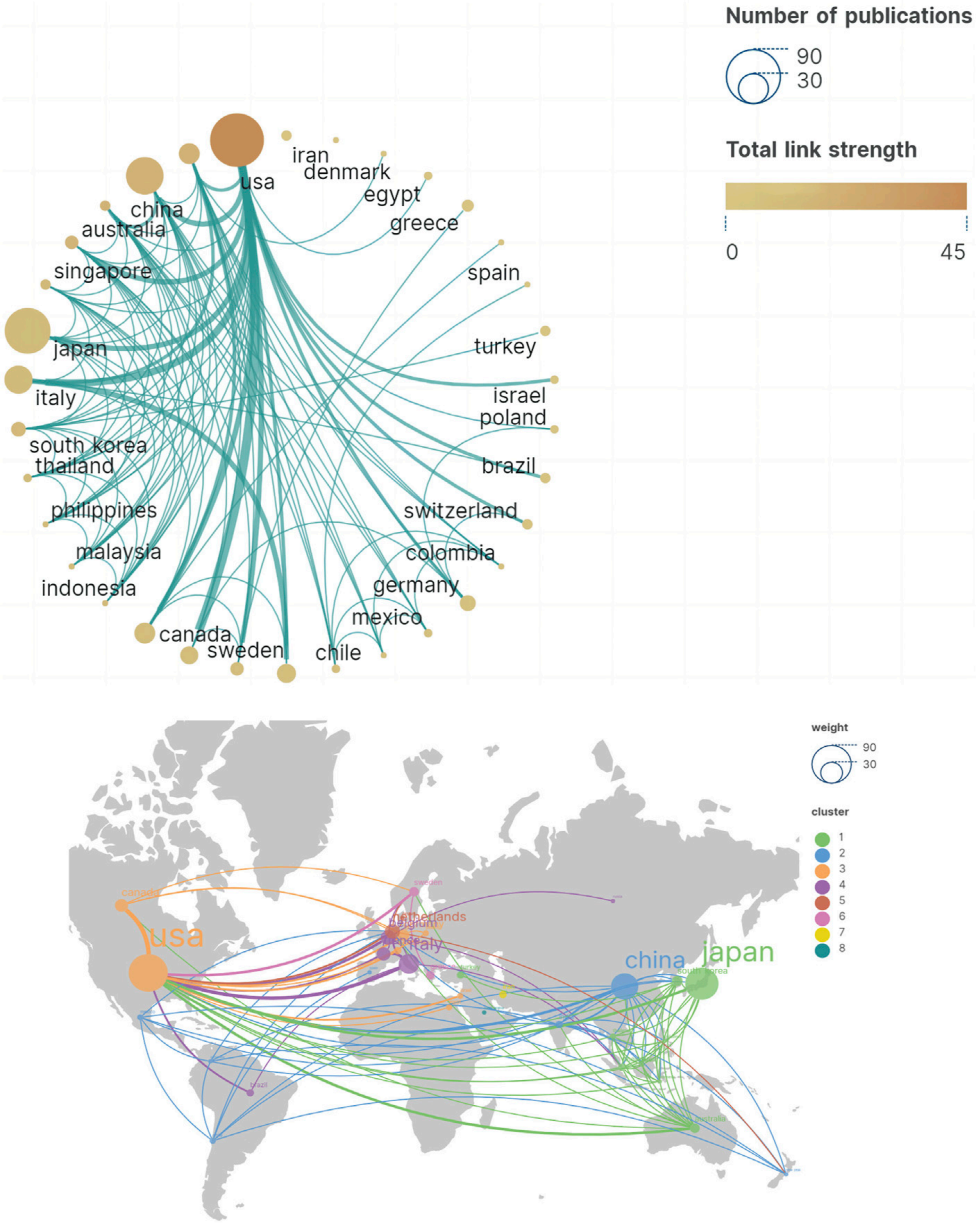
Cooperation map of countries/regions in refractory GERD.

In the past, 322 institutions from 36 countries and regions have conducted research and published articles in this field. Table 1 presents the 10 countries that have contributed the most papers, with the United States, Japan, and China leading with 74, 58, and 36 articles respectively. These publications account for a significant portion of the total articles (69.71%) and highlight their prominent role in the development of refractory GERD. Figure 3 visualize the cooperative relationships between countries. By comparing the centrality, it can be found that United States and Netherlands have the highest centrality, which is 0.4 and 0.36 respectively, indicating that these two countries have closer cooperation with other countries in this field, while other countries have less communication with each other. Table 2 describes the top 10 institutions with the largest number of papers published, and Japanese institutions account for the largest proportion, with 7 institutions. In addition, the single institution with the highest citation is the University of Arizona in the United States, with a citation frequency of 465 times, which represents the institution has a certain influence in this academic field.

**TABLE 1:** Top 10 countries/regions by number of publications.

Rank	Country	Publications	Centrality	Citations	TLS
1	United States	74 (30.71%)	0.40	2,011	43
2	Japan	58 (24.07%)	0.09	1,036	13
3	China	36 (14.84%)	0.04	380	19
4	Italy	22 (9.13%)	0.02	652	14
4	Belgium	12 (4.98%)	0.17	572	10
4	Netherlands	12 (4.98%)	0.36	920	19
5	France	10 (4.15%)	0.00	395	8
5	Canada	9 (3.73%)	0.01	455	10
6	United Kingdom	9 (3.73%)	0.09	390	14
7	South korea	6 (2.49%)	0.10	162	13

TLS: total link strength.

**TABLE 2:** Top 10 institutions by number of publications.

Rank	Organization	Publications	Citations	TLS
1	Nippon Medical School (Japan)	14	213	23
2	Gunma University Hospital (Japan)	9	223	58
3	Osaka City University (Japan)	8	230	44
3	Northwestern University (United States)	8	211	43
3	Tohoku University (Japan)	8	208	34
4	University of Arizona (United States)	7	465	80
4	Queen Mary University of London (United Kingdom)	7	374	23
4	Osaka Medical College (Japan)	7	173	54
4	Shimane University (Japan)	7	170	57
5	University of Padua (Italy)	6	193	26
5	University of Pisa (Italy)	6	193	23
5	Hamamatsu University School of Medicine (Japan)	6	132	31

TLS: total link strength.

### 3.3 Analysis of authors

**FIGURE 4:**
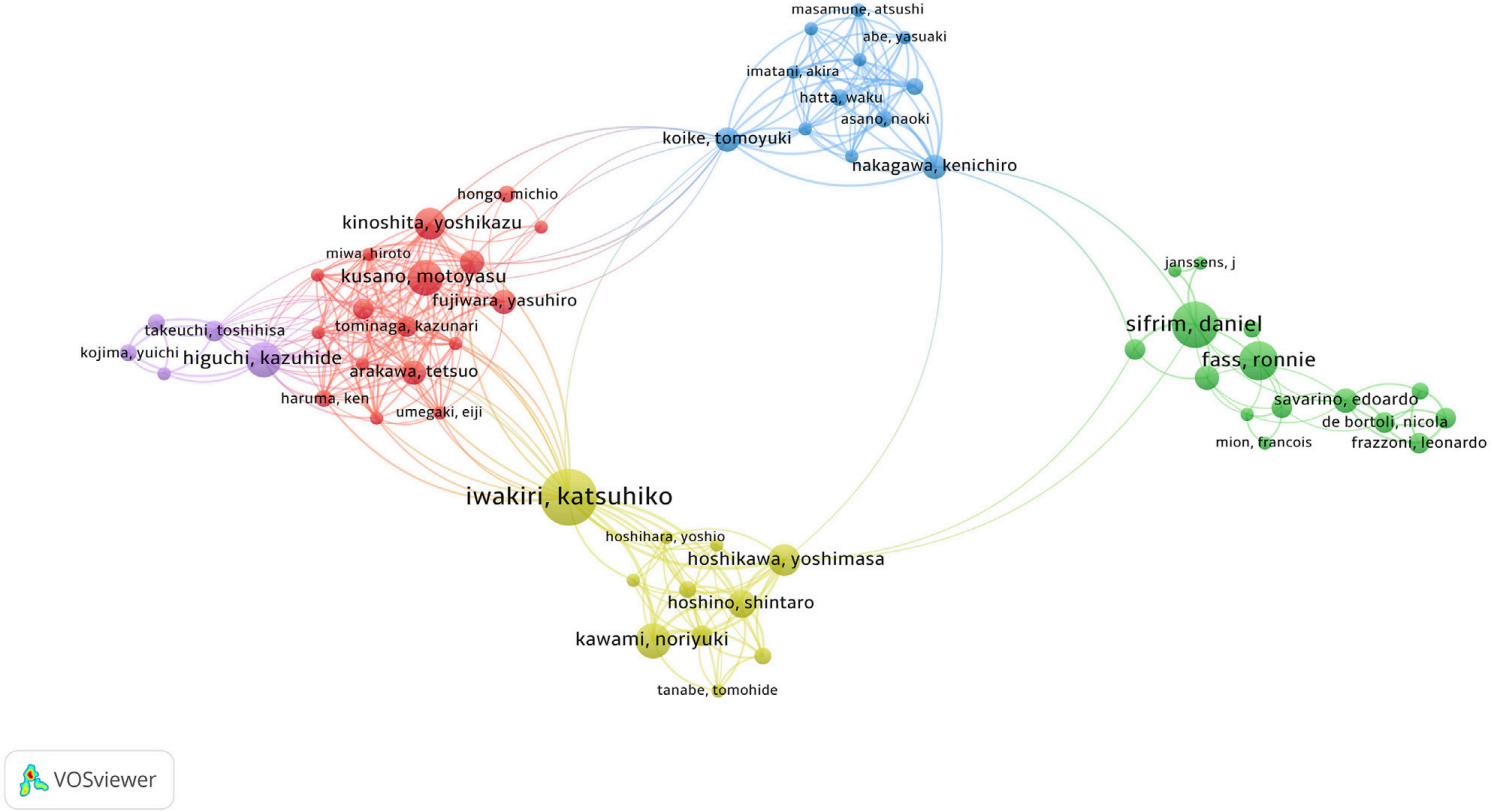
Cooperation map of authors in refractory GERD.

A total of 1,265 authors have contributed to the field of research Figure 4. The author cooperation network depicts different author groups or teams using distinct colors. Node size represents the number of papers, while line thickness indicates the strength of collaborative relationships among authors (Chen, 2006). Table 3 presents the top 10 authors by number of publications and their number of citations and H-index. Notably, Japanese scholars have made remarkable contributions to the research on refractory GERD. Among them, Professor Iwakiri and Katsuhiko stand out as not only having the highest number of publications, but also the closest cooperation with other authors. It is worth mentioning that Iwakiri, Katsuhiko, Danie Sifrim, Ronnie Fass, Nakagawa Kenichiro, Koike Tomoyuki, Kinoshita Yoshikazu, Higuchi Kazuhide, Hoshikawa Yoshimasa eight authors may be important leaders in the collaborative project between institutions. Furthermore, it is noteworthy that Professor Ronnie Fass holds the highest H-index value. The author with the highest number of citations is Professor Danie Sifrim. These findings underscore their significant academic stature and potential pivotal role in pioneering breakthroughs related to refractory GERD.

**TABLE 3:** The top 10 productive authors.

Rank	Authors	Country	Publications	Citations	H-index	TLS
1	Iwakiri, Katsuhiko	Japan	15	267	25	110
2	Sifrim, Daniel	United Kingdom	10	596	36	31
3	Fass, Ronnie	United States	10	499	62	13
4	Kusano, Motoyasu	Japan	9	223	28	53
4	Higuchi, Kazuhide	Japan	9	184	45	58
4	Kawami, Noriyuki	Japan	9	157	12	47
5	Kinoshita, Yoshikazu	Japan	8	181	43	46
5	Hoshikawa, Yoshimasa	Japan	8	130	9	47
6	Hoshino, Shintaro	Japan	7	104	20	44
7	Arakawa, Tetsuo	Japan	6	166	48	45

TLS: total link strength.

### 3.4 Analysis of journals and literature citation

The top 10 journals with the highest number of citations and their number of publications, impact factors, and Journal Citation Reports (JCR) are presented in Table 4, which can provide a reference for the quality assessment and selection of journals in this research area. The most cited journals are *GUT* and GASTROENTEROLOGY. Although 70% of the top 10 journals are in the JCR Q1 region, there are still many articles on refractory GERD published in journals with low impact factors, suggesting that more in-depth and high-quality research should be carried out. In terms of article quantity, *DIGESTION* emerges as the most popular journal among authors, with a total of 17 publications.

**TABLE 4:** The top 10 most cited journals.

Rank	Journal	Citations	Publications	If	H-index	JCR
1	*Gut*	742	5	24.5	262	Q1
2	*Gastroenterology*	513	5	29.4	368	Q1
3	*American Journal of Gastroenterology*	419	9	10.2	234	Q1
4	*Alimentary Pharmacology* *&* *Therapeutics*	384	12	7.6	159	Q1
5	*Neurogastroenterology and Motility*	288	10	3.5	93	Q2
6	*Digestion*	255	17	3.2	71	Q3
7	*Surgical Endoscopy and Other Interventional Techniques*	239	12	3.1	141	Q1
8	*Journal of Gastroenterology*	216	7	6.3	99	Q1
9	*Clinical Gastroenterology and Hepatology*	176	4	12.6	151	Q1
10	*Digestive Diseases and Sciences*	150	11	3.1	113	Q3

TLS: Total link strength IF: impact factor (Journal Citation Reports 2022).

Table 5 shows the top 15 cited articles among the 241 articles (Fass et al., 2000; Klinkenberg-Knol et al., 2000; Koek et al., 2003; Hemmink et al., 2008a; Fass and Sifrim, 2009a; Slaughter et al., 2011; Vela et al., 2011; Sifrim and Zerbib, 2012a; Tominaga et al., 2012; Noar et al., 2014; Penagini et al., 2015a; Fock et al., 2016; Hoshino et al., 2017a; Frazzoni et al., 2017; Spechler et al., 2019; Delshad et al., 2020). Among them, the article published by Klinkenberg-Knol (Klinkenberg-Knol et al., 2000) in 2000 was cited the most times, reaching 390 times. This study followed patients with refractory reflux esophagitis (RE) for an average duration of 6.5 years, providing substantial clinical evidence supporting the long-term efficacy and safety of omeprazole in treating this disease. It may have played a pivotal role in promoting the widespread clinical application of omeprazole for this condition. Among these top 15 cited articles, two were published within the past 5 years. One was a randomized controlled trial (Spechler et al., 2019) comparing surgery and medicine as treatment options for this disease, published in the NEW ENGLAND JOURNAL OF MEDICINE in 2019, with a total citation count of 121. Additionally, there was a large-scale cohort study published by Sean (Delshad et al., 2020) in GASTROENTEROLOGY in 2020 that revealed crucial epidemiological characteristics associated with refractory GERD.

**TABLE 5:** The top 15 cited articles related to refractory gastroesophageal reflux disease.

Rank	Title	Year	Journal	If	JCR	Types of research	Total Citations
1	Long-term omeprazole treatment in resistant gastroesophageal reflux disease: Efficacy, safety, and influence on gastric mucosa	2000	*Gastroenterology*	29.4	Q1	Clinical Trial	390
2	Diagnosis and management of patients with reflux symptoms refractory to proton pump inhibitors	2012	*Gut*	24.5	Q1	Review	224
3	Management of heartburn not responding to proton pump inhibitors	2009	*Gut*	24.5	Q1	Review	219
4	Effect of the GABA(B) agonist baclofen in patients with symptoms and duodeno-gastro-oesophagealreflux refractory to proton pump inhibitors	2003	*Gut*	24.5	Q1	Clinical Trial	190
5	Esophageal pH-Impedance Monitoring in Patients With Therapy-Resistant Reflux Symptoms: “On” or"Off ”Proton Pump Inhibitor?	2008	*American Journalof Gastroenterology*	10.2	Q1	RCT	154
6	Randomized Trial of Medical versus Surgical Treatment for Refractory Heartburn	2019	*New England Journal of Medicine*	158.5	Q1	RCT	121
7	Asia-Pacific consensus on the management of gastro-oesophageal reflux disease: an update focusingon refractory reflux disease and Barrett’s oesophagus	2016	*Gut*	24.5	Q1	Practice Guideline	109
8	Caution About Overinterpretation of Symptom Indexes in Reflux Monitoring for Refractory Gastroesophageal Reflux Disease	2011	*Clinical Gastroenterology and Hepatology*	12.6	Q1	Cross-Sectional Study	107
9	Prevalence of Gastroesophageal Reflux Disease and Proton Pump Inhibitor-Refractory Symptoms	2020	*Gastroenterology*	29.4	Q1	Cohort Study	88
10	The added diagnostic value of postreflux swallow-induced peristaltic wave index and nocturnal baseline impedance in refractory reflux disease studied with on-therapy impedance-pH monitoring	2017	*Neurogastroenterologyand Motility*	3.5	Q2	Cohort Study	87
11	Refractory Heartburn: Comparison of Intercellular Space Diameter in Documented GERD vs. Functional Heartburn	2011	*American Journal of Gastroenterology*	10.2	Q1	Comparative Study	70
12	Rikkunshito improves symptoms in PPI-refractory GERD patients: a prospective, randomized, multicenter trial in Japan	2012	*Journal of Gastroenterology*	6.3	Q1	RCT	70
13	Omeprazole 40 mg once a day is equally effective as lansoprazole 30 mg twice a day in symptom control of patients with gastro-oesophageal reflux disease (GERD) who are resistant to conventional-dose lansoprazole therapy - a prospective, randomized, multi-centre study	2000	*Alimentary Pharmacology* *&* *Therapeutics*	7.6	Q1	RCT	69
14	Long-term maintenance effect of radiofrequency energy delivery for refractory GERD: a decade later	2014	*Surgical Endoscopy and Other Interventional Techniques*	3.1	Q1	Clinical Trial	68
15	Efficacy of Vonoprazan for Proton Pump Inhibitor-Resistant Reflux Esophagitis	2017	*Digestion*	3.2	Q3	Clinical Trial	66

TLS: Total link strength IF: impact factor (Journal Citation Reports 2022).

### 3.5 Analysis of references with citation bursts

**FIGURE 5:**
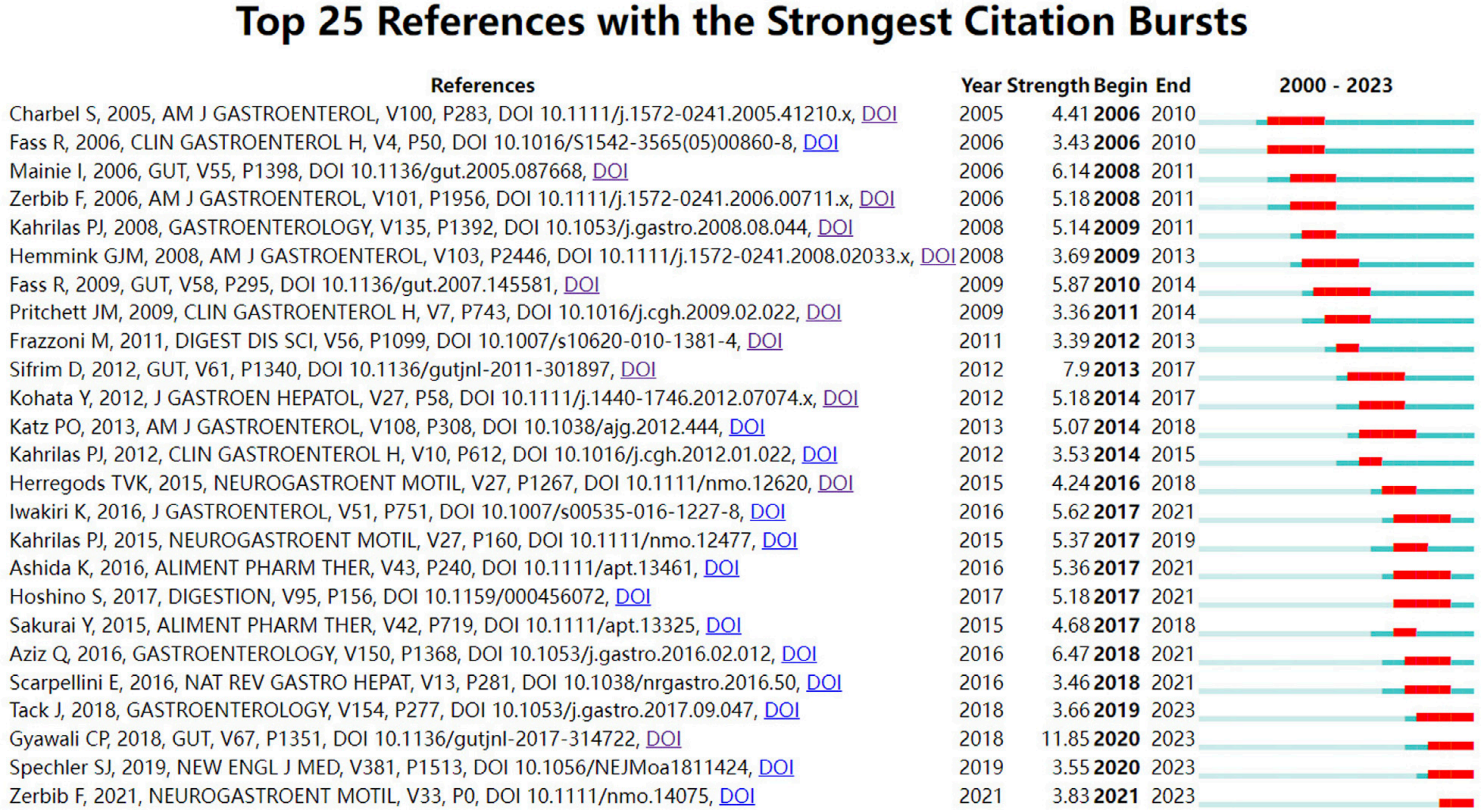
References with the strongest citation bursts.

Figure 5 shows the top 25 references with the strongest citation bursts from 2000 to 2023 (Charbel et al., 2005; Mainie et al., 2006; Vakil et al., 2006; Zerbib et al., 2006; Kahrilas et al., 2008a; Hemmink et al., 2008b; Dent et al., 2008; Fass and Sifrim, 2009b; Sweis et al., 2010; Frazzoni et al., 2011a; Sifrim and Zerbib, 2012b; Kahrilas et al., 2012; Kohata et al., 2012; Katz et al., 2013b; Kahrilas et al., 2014; Herregods et al., 2015; Kahrilas et al., 2015; Sakurai et al., 2015; Ashida et al., 2016; Aziz et al., 2016; Iwakiri et al., 2016; Scarpellini et al., 2016; Hoshino et al., 2017b; Gyawali et al., 2018a; Tack and Pandolfino, 2018; Zerbib et al., 2021a). Among these, 12 were reviews or consensus documents that guided subsequent research endeavors. Burst literature is identified using Professor Kleinberg’s burst detection algorithm, which highlights references that have been frequently cited in a short period and labeled as hotspots (Chen et al., 2012). The red line segment on the right corresponds to the burst time interval of each reference, while strength indicates its intensity. The Lyon consensus, issued by global gastroenterologists in 2018, had the highest outbreak intensity (Gyawali et al., 2018b) and continues to be widely recognized and frequently cited by peers.

### 3.6 Analysis of co-occurrence keywords

**FIGURE 6:**
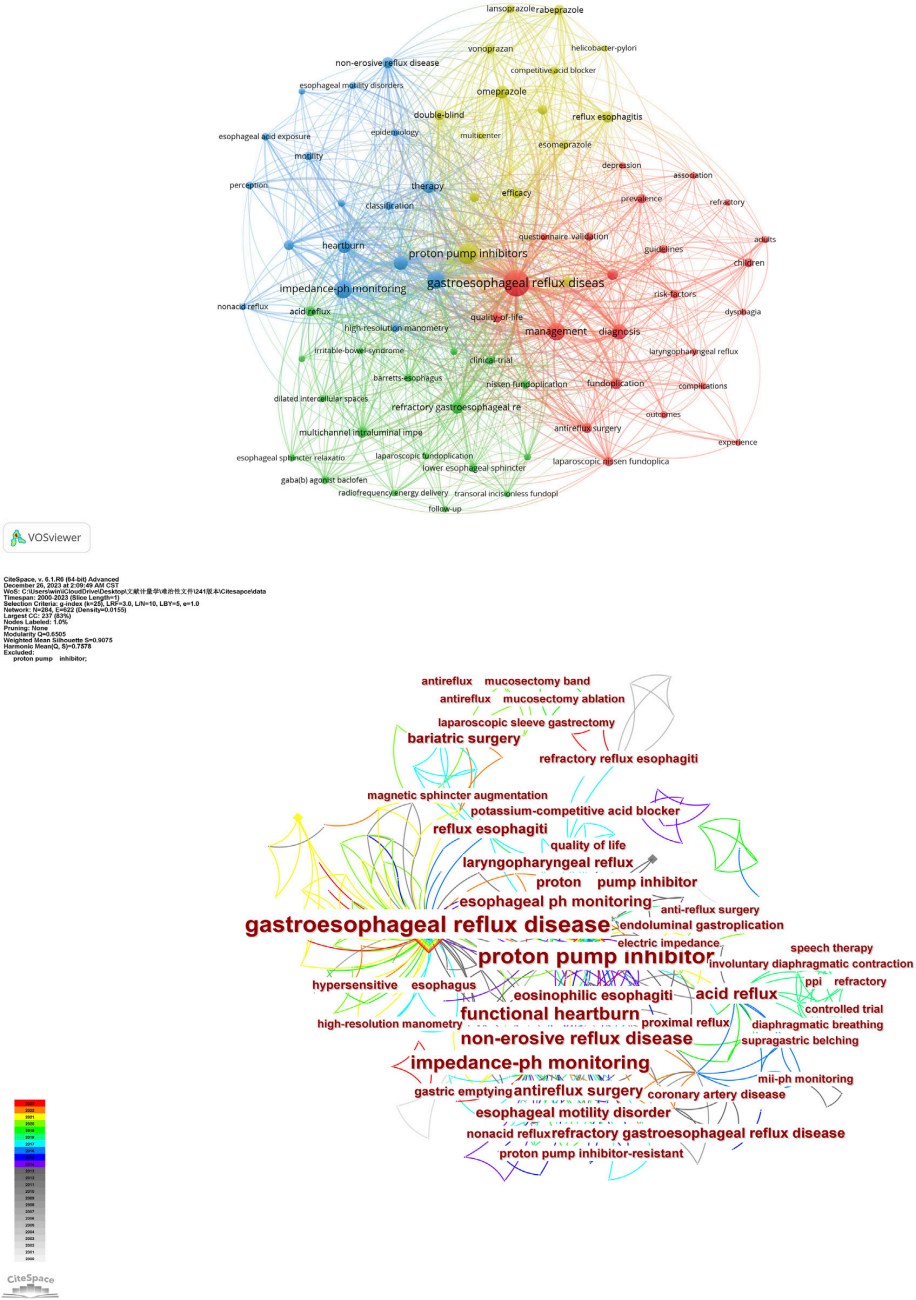
Map of keyword clustering in refractory GERD.

Keywords play a crucial role in quickly identifying the literature’s topic and effectively extracting vital information (Dotsika and Watkins, 2017). Keywords co-occurrence, an essential component of bibliometric analysis, aids in speculating potential research hotspots. In this study, we extracted 74 keywords with the highest co-occurrence and presented them as a network in Figure 6. Employing a clustering algorithm, we divided these keywords into four clusters distinguished by colors: red, yellow, blue, and green. The red cluster pertains to refractory GERD, focusing on its management and diagnosis. Key terms include “management,” “diagnosis,” “guidelines,” and “prevalence.” The green cluster primarily explores the mechanism behind refractory GERD using Keywords such as “mechanism,” “acid reflux,” “esophageal sphincter relaxation,” and “GABA(B) agonist baclofen.” This module aims to identify possible causes for this challenging condition. The yellow cluster delves into drugs that suppress gastric acid like PPIs, omeprazole, rabeprazole, potassium-competitive acid blockers (P-CABs), and vonoprazan. This module mainly discusses the application and development of drugs represented by PPIs. The blue cluster mainly deals with refractory symptoms and detection methods. The main keywords are “refractory symptoms,” “non-erosive reflux disease,” “impedance-ph monitoring,” and “high-resolution manometry.” This cluster discusses the characteristics of refractory GERD and the application of new detection methods such as esophageal impedance-pH monitoring and high-resolution manometry in this field. The topics corresponding to the four clusters covered the mainstream academic literature on physiology and pathology, diagnosis, detection methods, and treatment of refractory GERD.

**FIGURE 7:**
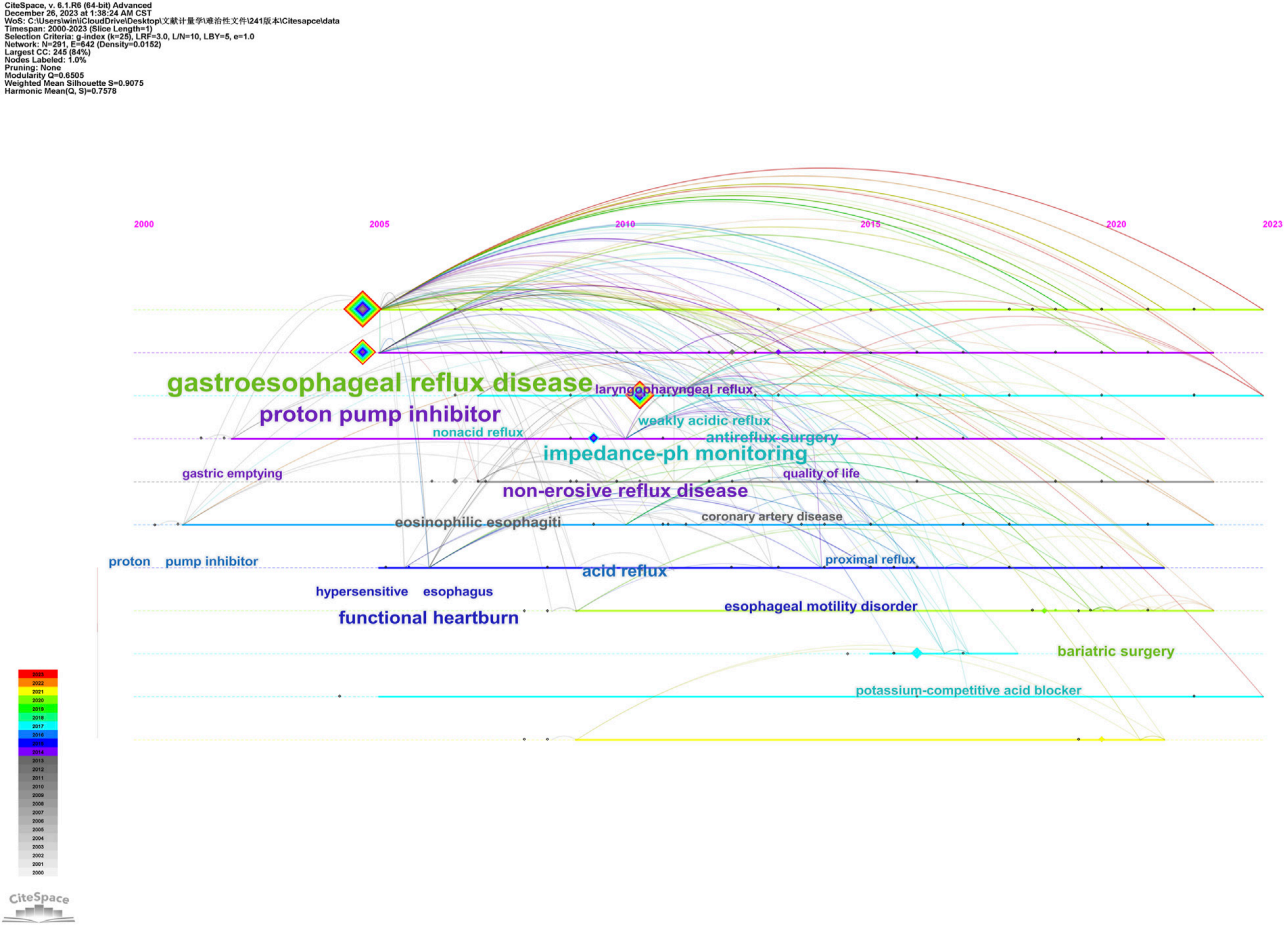
Timeline view and evolutionary path in refractory GERD.

Furthermore, Figure 7 illustrates the temporal evolution of keywords throughout the study period using years as a reference point. It is evident that post-2005, researchers shifted their focus toward understanding the mechanism and identification of this disease. Keywords included “gastric emptying,” “eosinophilic esophagitis,” “hypersensitive esophagus,” and “functional heartburn.” Around 2010, researchers developed an interest in exploring the nature of refluxate in refractory gerd with subject words such as “nonacid reflux,” “acid reflux,” and “weakly acidic reflux.” By 2015, attention was directed towards investigating the role of esophageal motility disorders with keywords like “esophageal motility” and “proximal reflux.”

## 4 Discussion

This work utilized a diverse range of bibliometric tools to analyze refractory GERD across five dimensions: publication trends, country and author contributions, core literature analysis, hot topics investigation, and frontier direction exploration. The main conclusions are as follows.

### 4.1 General information

In the more than 20 years since 2000, clinicians and researchers have shown significant interest in refractory GERD. Consequently, related research has been carried out rapidly in different regions, and related articles have been increasing year by year.

For the surge in publications on refractory GERD since 2011, we analyzed several reasons. First, epidemiological surveys published in 2009–2010 revealed that the persistence of reflux symptoms after taking PPIs was very common, ranging from 17% to 32%. Moreover, this incidence was observed to be increasing over time, prompting researchers to direct their attention towards this concerning phenomenon that posed challenges for both patients and physicians (El-Serag et al., 2010a; Chey et al., 2010). Secondly, the International High Resolution Esophageal Manometry Group published the first edition of the classification standard for esophageal motility disorders in 2009, which was subsequently named Chicago Classification (CC), heralding a groundbreaking advancement in esophageal manometry technology. Peter J invented pressure topography plots based on traditional conventional manometric recordings, which are superior in defining the spatial characteristics of esophageal constriction segments and in determining pressure changes (Kahrilas, 2010). As a result, HRMs that incorporate this technology can accurately identify patients with more subtle esophageal motility disorders and categorize them according to classification criteria as distal esophageal spasm, vigorous achalasia, functional obstruction, and subtypes of nutcracker esophagus. This change has transformed esophageal manometry data from crude to refined and the interpretation of results from complex to intuitive. The new technique facilitates clinicians to detect anatomical defects of the esophagus as well as other dysfunctions, thus distinguishing truly refractory patients, which has a significant impact on the rigorous screening of eligible cases for subsequent clinical studies and convincing statistics (Kahrilas et al., 2008b). Meanwhile, the method developed by L. B. Gerson et al., in 2011 offers a novel approach to assess clinical features, severity, and predict the necessity of additional anti-reflux treatment. This advancement holds significant potential for enhancing the management of refractory GERD in primary care and community settings (Gerson et al., 2011). As a result, on the one hand, there is a growing awareness among individuals regarding the limitations of PPIs therapy, and an increasing number of clinicians are reporting refractory patients who exhibit resistance to acid suppressors. On the other hand, newly discovered detection and evaluation methods have provided directions for subsequent research, thereby prompting researchers to undertake numerous in-depth studies on the etiology of refractory cases. It is highly plausible that these factors have contributed to the surge in publications on refractory GERD since 2011.

The United States, being the country with the highest number of publications in this field, has played a leading role in the development of research on refractory GERD. This finding may be attributed to researchers’ attention to the high incidence and substantial medical burden associated with this condition in the United States (Wahlqvist et al., 2006; El-Serag, 2007; El-Serag et al., 2010b; Delshad et al., 2020; Nirwan et al., 2020; Howden et al., 2021). Although Japan ranks second in total publications, it accounts for 7 out of the top 10 institutions, indicating the significant prominence of refractory GERD within Jap anese academic circles, possibly related to the country’s advanced endoscopic technology and public health awareness regarding early esophageal cancer screening (Pathirana and Poston, 2001; Dobashi et al., 2022; Fujishiro and Matsumoto, 2022). The United States and the Netherlands exhibit closer collaborations with other countries, serving as a valuable example for other countries to enhance cooperation and exchange to broaden research ideas.

Potential reasons for the leading position of these countries and institutions include the following. The first reason is related to social and demographic factors. A study showed that refractory GERD patients in the United States have the highest proportion among all the countries and regions surveyed, which accounts for 54.1% of the patients with GERD (Delshad et al., 2020). This has a serious adverse impact on the quality of life and work and increases the economic burden, but also triggers the attention and thinking of clinicians and researchers. Secondly, the United States has made significant contributions to the advancement of detection technology through the invention and development of impedance-pH monitoring and high-resolution manometry. Meanwhile, Japan’s contribution lies in pioneering gastric fiberscope prototypes, which have greatly facilitated the progress of electronic endoscopy. These remarkable achievements have served as catalysts for extensive research conducted by various institutions and scholars.

In the 1950s, Charlie at Mayo Clinic pioneered the systematic study of esophageal motility, and led to the landmark discovery of the Lower Esophageal Sphincter (LES) (Code et al., 1956). Later, many American gastroenterologists focused on esophagology and became prominent esophagologists, including Donald Castell, who was called the “Pope of Esophagology.” He took the initiative to establish a multidisciplinary collaboration with Radiology and cardiothoracic surgery, forming a cohesive team. He initiated multidisciplinary collaborations including esophageal, radiology, and cardiothoracic surgery, as well as establishing close contact with researchers from Japan and Australia. Additionally he innovatively integrated radiologic imaging with manometry techniques to clarify esophageal muscle function and summarized various concepts pertaining to esophageal motility disorders that hold significant value for further investigation. Such as ineffective esophageal motility (IEM) (Leite et al., 1997), achalasia (Olsen et al., 1957), TLESRs (Dent et al., 1980). He also collaborated closely with Tom DeMeester, a cardiothoracic surgeon, who proposed the DeMeester score for assessing reflux events and acid exposure. This contribution played a significant role in the subsequent advancement of surgical techniques for refractory GERD (Grubic and Crookes, 2021). We can gain valuable insights from their successful experience through the aforementioned development process. Multi-disciplinary treatment (MDT) and multi-regional and institutional collaboration serve as the driving forces for advancing progress in the research field. This model provides experts with the opportunity to discuss the issues they face and express different views, as well as the possibility of innovation. In addition, the rapid development of modern science and technology is also crucial to the impact of this disease. Endoscopic technology, pressure transducer technology, and the pressure topography plots mentioned above are the technical support and guarantee for progress.

In terms of authors, Professor Iwakiri Katsuhiko has the highest number of publications and collaborates most closely with other authors. The author with the highest H-index is Professor Ronnie Fass. The most highly cited author is Professor Daniel Sifrim, who has expertise in neurogastroenterology and motility and pathophysiology of GERD. These three distinguished authors hold prominent positions within this domain and are poised to contribute significantly towards groundbreaking discoveries in refractory GERD. The most cited journals are *GUT* and *GASTROENTEROLOGY*, while *DIGESTION* has emerged as a popular choice among authors for publication purposes. This information can serve as a valuable reference for assessing journal quality and selecting appropriate outlets within this specialized field.

Although the number of article citations is influenced by publication length, it is undeniable that highly cited articles serve as the foundation for subsequent advancements in the field. These articles encompass valuable knowledge worth learning and the key issues that subsequent researchers are concerned about. From the distribution of the top 15 cited literature types in this study, clinical trials accounted for 80%, and the remaining 20% were consensus and reviews.

Clinical trials primarily focused on the efficacy evaluation and long-term follow-up results of drug interventions and surgical treatments. And the accurate identification of refractory symptoms, the clinical value of new detection methods, and their parameters. However, there remains a scarcity of highly cited basic research in this field.

From the evolution of keywords over time, we can see that researchers’ thoughts and strategies are interestingly divided into two different directions. A portion of the group spent a lot of effort upfront (probably before 2010) in the study of the efficacy of PPIs in refractory GERD, and the exploration of the mechanisms of acid reflux. Consequently, they continue to advocate for acid inhibition therapy as the preferred strategy. Their focus lies in enhancing the efficiency of acid inhibition, developing novel and more potent acid inhibition agents, accurately identifying refractory individuals who would benefit from strong acid inhibition therapy, and improving the duration during which stomach pH levels are maintained above 4.

With the researchers’ further investigation into the pathogenesis of refractory GERD and the persistence of acid inhibition therapy failure, another part of people are gradually recognizing the intricate multifactorial mechanisms involved. Consequently, their interest and patience in acid inhibition therapy for refractory patients who exhibit resistance to acid suppression medications have waned. They have then shifted their focus to intervening in various alternative mechanisms, such as physiological acid or weakly acidic reflux events, persistent mucosal microscopic damage (dilated intercellular spaces), chemical clearance ability, ineffective esophageal motility, TLESRs, visceral hypersensitivity, neuroimmune-mediated responses, psychological factors (stress/anxiety and hypervigilance). The objective is to utilize these emerging mechanisms for the development of innovative pharmacological and non-pharmacological therapies encompassing behavioral interventions, endoscopy, and surgery.

### 4.2 Standardized diagnosis and management

The definition of refractory GERD has been a subject of controversy, primarily concerning the treatment regimen and daily dosage of PPIs, as well as the criteria for recognizing ineffectiveness or poor response. In 2006 (Richter, 2006), refractory GERD was defined in a study as the absence of significant symptom improvement after 4–8 weeks of twice-daily PPIs treatment. The 2009 guideline (Fass and Sifrim, 2009b) published by Ronnie Fass and Daniel Sifrim defines refractory GERD as the lack of response to once-daily treatment with PPIs. According to the 2012 guidelines (Sifrim and Zerbib, 2012b) for managing refractory GERD, persistent symptoms of regurgitation or heartburn occurring at least three times per week are observed after more than 12 weeks of treatment with double-dose PPIs.

Since then, researchers have gradually realized that the determination of “refractory” status is largely influenced by patients’ subjective perceptions and treatment expectations rather than objective assessments. In 2021, the European Society for Neurogastroenterology and Motility (ESNM) and the American Neurogastroenterology and Motility Society (ANMS) (Zerbib et al., 2021b) have made significant advancements in distinguishing subjective symptoms from objective evidence by proposing three definitions: refractory GERD, refractory reflux-like symptoms, and refractory GERD symptoms. Among them, refractory GERD is defined as objective evidence of GERD that has not disappeared after standardized drug treatment, including (erosive esophagitis, abnormal esophageal acid exposure and/or elevated numbers of reflux episodes on reflux monitoring performed on therapy). The advantage of this classification is that it can change the basis for judging whether the disease is “refractory” or not from the patient’s subjective feeling to the objective indicators derived from the clinician’s specialized examination, which can help to strictly differentiate the patients with truly refractory GERD, and make more accurate judgments of the patient’s condition. This way of defining may be the trend in the future.

### 4.3 Evolution and characteristics of different drugs

Based on the specificity of the definition of refractory GERD, we need to take into account the changes in physiopathology caused by clinical treatment before the diagnosis of this disease in this population. It has been shown that erosive oesophagitis healing rates can be as high as 90% after 8 weeks of PPIs or vonoprazan treatment (Xiao et al., 2020). The fact that the patient was already receiving regular acid-suppressive therapy prior to the discovery of refractoriness may have led to the healing of the eroded esophageal mucosa, which may explain the following two findings. The first notable observation is the high prevalence of non-erosive reflux disease (NERD) among patients with refractory GERD (Kim et al., 2015). A second finding reveals that individuals with NERD exhibit lower rates of symptomatic response to PPIs compared to those with RE (Martinez et al., 2003). In addition, the strong acid-inhibiting effect of acid-suppressive drugs may affect the acidic reflux of pH monitoring in patients. Marcelo (Vela et al., 2001) compared esophageal impedance-pH monitoring results in 12 patients with GERD before and after omeprazole treatment and found that the proportion of acid reflux decreased from 45% to 3% before treatment, while the proportion of non-acidic reflux (pH ≥ 4) reflux increased from 55% to 97%. Furthermore, numerous studies have documented the presence of weakly acidic reflux outside of the normal range in patients with refractory GERD (Mainie et al., 2006; Sharma et al., 2008; Frazzoni et al., 2011b; Kawami et al., 2018). Radu (Tutuian et al., 2008) conducted a study in patients with persistent symptoms despite acid suppressive therapy and observed 3,547 reflux events, of which 84.3% were non-acidic. However, it is unclear whether the high percentage of non-acidic reflux is attributable to the treatment or represents unique pathological manifestations associated with the condition and its possible role in the development and progression of refractory GERD. The causality between the high rate of non-acidic reflux and acid-suppressive treatment remains uncertain, as well as its potential role in the pathogenesis and progression of this disease.

Insufficient acid inhibition is one of the underlying mechanisms contributing to this disease, and researchers have turned to replacing potent acid inhibition agents as a potential solution. Since its initial global approval in Japan (Garnock-Jones, 2015) on 26 December 2014, Vonoprazan has emerged as a recommended treatment for refractory GERD due to its superior efficacy in suppressing acid production and promoting esophageal mucosal healing compared to PPIs (Miwa et al., 2019; Tanabe et al., 2019; Ochiai et al., 2021; Xu et al., 2023). Meanwhile, it exhibits no susceptibility to variations in CYP2C19 genotype (Kagami et al., 2016). However, a retrospective analysis of a small sample revealed that even with double doses of P-CAB, some NERD patients still exhibited poor responses. In comparison to the effective group, the ineffective group showed an increased proportion of 4 ≤ pH ≤ 5, along with a decrease in acid exposure time (AET). And researchers propose that a pH of 5 may be the threshold for influencing symptom onset (Abe et al., 2021). In Noriyuki Kawami’s study (Kawami et al., 2018), all 42 double-dose P-CAB-resistant NERD patients without esophageal motility disorders had no detectable abnormal acid exposure, and 41.9% of these patients were SI-positive, all with weakly acidic reflux. The possible mechanism of refractory GERD is now more clear. Weakly acidic reflux events and the amplificated sensitivity of the esophagus to refluxants play a crucial role in symptom occurrence, making further acid inhibition an unwise choice for symptom relief.

In response, researchers have attempted a therapeutic shift from antiacid to antireflux therapy, focusing on the transient lower oesophageal sphincter relaxations (TLESRs), a key pathogenesis of refractory GERD (Hershcovici et al., 2011). Baclofen, a gamma-aminobutyric acid (GABA)-B receptor agonist, has been found to effectively reduce TLESRs (Koek et al., 2003). A recent randomized, double-blind, placebo-controlled study (Pauwels et al., 2022; Raymenants et al., 2022) found that Baclofen could significantly reduce SAP positivity, that is, symptoms associated with reflux episodes. However, its clinical application is hindered by the side effects resulting from its ability to penetrate the blood-brain barrier (Kent et al., 2020). Another placebo-controlled cross-over study (Sawada et al., 2020) reported that ONO-8539, an E-type prostanoid 1 receptor antagonist, inhibits TLESRs. These findings suggest that EP1 receptor may be a potential target for the treatment of refractory GERD.

The efficacy and benefit of gastrointestinal prokinetics in this condition remain uncertain. One perspective (Ishimura et al., 2015) suggests that Acotiamide has minimal impact on esophageal body contractions or EGJ compliance in both patients with GERD and healthy individuals. However, other studies (Yamashita et al., 2015; Yamashita et al., 2019) have indicated that Acotiamide can reduce TLESRs, improve esophageal bolus clearance in healthy individuals, alleviate persistent symptoms of refractory NERD, and decrease total reflux episodes. These episodes include acid reflux, proximal reflux, and liquid reflux. A recent meta-analysis (Jung et al., 2021) suggested that combining prokinetics with PPIs is more effective than using PPIs alone.

### 4.4 Advantages and disadvantages of surgery

For reflux events, surgeons tend to focus on the anatomical structure and physiological motor function of the esophagus to remodel the antireflux barrier. In the 1850s, Allison (Allison, 1951) first reported hiatal hernia repair, while Rudolf Nissen (Nissen, 1956) invented fundoplication, which initiated surgical intervention for GERD. In 1991, antireflux surgery (Dallemagne et al., 1991) entered the laparoscopic era. The development trend now leans towards more minimally invasive and readily accepted endoscopic antireflux surgery. Since the emergence of antireflux surgery, addressing symptoms that are unresponsive to medical treatment or severe esophagitis has become a primary concern. Anti-reflux mucosectomy (ARMS) and radiofrequency energy delivery (STRETTA), as emerging endoscopic antireflux procedures, have demonstrated comparable clinical efficacy. The former inhibits reflux by inducing scar contractures in the damaged cardiac mucosa, making it a minimally invasive and effective treatment for refractory GERD. The 270 ARMS is recommended for reducing the incidence of postoperative dysphagia (Sumi et al., 2021; Yang et al., 2022; Zhang et al., 2022).

The latter is proven to induce LES muscle remodeling through stimulation, thereby reducing the occurrence of TLESRs and esophageal acid exposure. During a follow-up period of 10 years after surgery, 72% of patients with refractory GERD returned to a normal quality of life, and 64% reduced their use of PPIs by at least half (Noar et al., 2014). It is recommended as the primary choice for endoscopic treatment following unsuccessful fundoplication (Katz et al., 2013c; Wang Y. et al., 2023).

Additionally, a range of surgical options, such as transoral incisionless fundoplication (TIF) and magnetic sphincter augmentation (MSA), are available to alleviate refractory symptoms (McCarty et al., 2018; Roark et al., 2020; Kalapala et al., 2022; Patel et al., 2022). However, most of the current exploration of surgical interventions for this condition is based on retrospective studies and case reports. Randomized controlled trials and long-term follow-up, as well as the development of personalized surgical recommendations for refractory GERD, are the focus of future research.

It is important to acknowledge that a high level of confidence in the diagnosis of refractory GERD is crucial before considering any invasive surgical intervention. The ICARUS guidelines highlight the necessity of preoperative esophageal manometry and impedance-pH monitoring if endoscopy is negative. The results of high-quality randomized, controlled trials (Spechler et al., 2019) in 2019 suggested that surgery is superior to medical therapy for patients with truly established refractory GERD. In September 2023, the Lyon consensus 2.0 recommended a switch to surgery in patients with refractory GERD who had both AET > 4% and more than 80 reflux episodes (Spechler et al., 2019; Gyawali et al., 2023).

Visceral hypersensitivity is a complex mechanism mediated by multiple factors that have been suggested to be potentially involved in the development of refractory GERD, including the expression of acid-sensing ion channels, localization of sensory nerve, as well as interactions between inflammatory mediators and neurotransmitters (Guarino et al., 2010; Ustaoglu et al., 2021; Ustaoglu and Woodland, 2023). A study by Rohof (Rohof et al., 2014) found that patients with refractory GERD had more proximal reflux than patients who responded to PPIs. Philip (Woodland et al., 2015) compared the mucosal integrity of the proximal and distal esophagus in healthy subjects and localized calcitonin gene-related peptide (CGRP)-immunoreactive nerve fibers and protein gene product (PGP) 9.5 immunoreactivity in nerve fibers and found that mucosal integrity was essentially the same at both ends, but that the proximal mucosa had more superficial afferent nerves. This unique feature provides anatomical evidence for proximal esophageal hypersensitivity. This finding is supported by a previous study conducted by Radu (Tutuian et al., 2008), which demonstrated a significant correlation between proximal reflux and the onset of symptoms in patients with refractory GERD, irrespective of whether the refluxants were acidic or non-acidic. Furthermore, esophageal hypervigilance may independently contribute to symptom perception in individuals with GERD (El-Serag et al., 2010b; Guadagnoli et al., 2021).

### 4.5 Novel mechanisms such as neuroimmune interaction

In recent years, with the advancement of research in neurology and immunology, the interaction between neuroimmunity has attracted the attention of researchers, particularly in peripheral organs such as the gastrointestinal tract. It has been reported that immune activation interacts with various gastrointestinal disorders, and visceral hypersensitivity may be associated with the stimulation of sensitive neurons by other algogenic mediators secreted by immune cells (Vanuytsel et al., 2023; Argüero and Sifrim, 2024; Leech and Peiris, 2024). The mast cells, which are part of the immune system, exhibit sensitivity to the endogenous microenvironment of immune cells. They induce sensitization of peripheral nerve function by releasing neuropeptides, such as histamine and other nociceptive mediators that act as pain inducers (Rosa and Fantozzi, 2013; Gupta and Harvima, 2018; Leech and Peiris, 2024). Ustaoglu’s study revealed an upregulation of nerve growth factor (NGF) expression in mast cells infiltrating the esophageal mucosa of patients suffering from reflux esophagitis (Ustaoglu et al., 2023). NGF plays a pivotal role in the development of chronic pain by binding to tyrosine kinase receptor A (NTRK1) on nerve fibers, thereby augmenting their numbers and eliciting hypersensitive pain sensations (Dothel et al., 2015; Eskander et al., 2015; Gupta and Harvima, 2018). Additionally, they assessed the association between mast cells and deep afferent nerve endings and discovered that these two are closely juxtaposed within the papillary structure of the esophageal mucosa, potentially contributing to hypersensitivity responses. They suggested that topical NGF antagonists could be a prospective therapeutic option for refractory GERD (Ustaoglu et al., 2023). The abundance of neuroimmune-related receptors offers diverse possibilities for selecting therapeutic targets.

Spechler’s team hypothesized (Souza et al., 2009) as early as 2009, following esophagoduodenostomy in rats and *in vitro* studies, that the tissue damage observed in reflux esophagitis may not be attributed to chemical corrosion caused by stimulation from refluxed gastric juice. Instead, it is more likely induced by acidified bile salts stimulating esophageal epithelial cells to secrete chemokines (IL-8 and IL-1β) and promoting inflammation. To further substantiate this hypothesis (Dunbar et al., 2016), a recent clinical study involving GERD patients with recurrent esophageal mucosal erosion after PPIs withdrawal found that the predominant inflammatory cells infiltrating the esophagus were T lymphocytes rather than neutrophils. Surface cell loss did not occur immediately but was observed subsequent to basal cell and papillary hyperplasia, providing evidence in support of a cytokine-mediated pathogenesis. Subsequent experiments have demonstrated (Huo et al., 2017; Souza et al., 2017) the crucial involvement of HIF-2α in RE pathogenesis, as it is activated by acidified bile salts and subsequently amplifies NF-kB/p65 activity, thus facilitating proinflammatory cytokine synthesis.

### 4.6 Considerations for various detection techniques

The Lyon Consensus, published in 2018, is based on the 2004 Porto (Sifrim et al., 2004) Consensus and, for the first time, puts forward the key recommendation based on a large number of clinical studies: impedance-pH monitoring is the “gold standard” for diagnosing GERD. The consensus also focuses on new parameters such as post-reflux swallow-induced peristaltic wave (PSPW), mean nocturnal baseline impedance (MNBI), and enriching objective detection indicators for refractory GERD. PSPW and MNBI are parameters that reflect the chemical clearance ability of the esophagus and the integrity and permeability of the esophageal mucosa, respectively. These two parameters have been shown to better identify pathological reflux (Wu et al., 2022) in patients with refractory reflux symptoms and can be used as characteristic indicators of refractory GERD (Frazzoni et al., 2023).

Clinicians are faced with two choices before recommending patients for endoscopy and impedance-pH monitoring, on or off PPIs? Lyon consensus 2.0 provides the answer; for patients with refractory GERD who have a previous diagnosis based on objective evidence, it is recommended that impedance-pH monitoring be performed during PPIs therapy, which can help to predict the outcome of surgery based on the regurgitation results (Gyawali et al., 2021). Conversely, if there is no objective diagnostic basis, impedance-pH monitoring after discontinuation of PPIs is recommended (Hemmink et al., 2008a; Katz et al., 2022). In addition, simple pH monitoring without impedance function is also recommended after withdrawal to rule out the effect of PPIs on acid reflux (Sifrim and Zerbib, 2012b). Considering the high rate of esophageal mucosal healing with antiacid drugs, the American College of Gastroenterology (ACG) suggests (Katz et al., 2022) that diagnostic endoscopy should be conducted 2–4 weeks after withdrawal PPIs for maximizing the accuracy of diagnosis of refractory GERD.

According to previous researches (Gawron et al., 2012), up to 42% of patients with refractory GERD continue taking PPIs despite negative findings on endoscopy and impedance-pH monitoring. This has prompted researchers to consider whether patients with refractory GERD can be terminated from long-term use of PPIs. A recent clinical trial (Yadlapati et al., 2021) has suggested that the number of days of AET ≥4% can be used to determine the necessity of continuing PPIs in patients with endoscopy-negative refractory GERD through prolonged wireless reflux monitoring. However, with the limitations of the paucity of relevant data and the fact that prolonged wireless reflux monitoring is not widely available in clinical practice, this issue remains to be resolved.

While impedance-pH monitoring is widely recommended as a reliable diagnostic tool, it has been regarded by certain scholars as a delicate examination susceptible to temporal variations. Roberto (Penagini et al., 2015b) performed prolonged wireless pH monitoring in 50 AET-negative patients with refractory heartburn, and found that half of them exhibited positive acid exposure after the second or third day. In a study by Yadlapati (Yadlapati et al., 2019), patients who did not respond to PPIs showed three distinct acid exposure trajectories during prolonged pH monitoring. Stephen (Hasak et al., 2020) found that the results of AET varied from day to day in patients with refractory GERD, with dominant AET patterns from long-term wireless pH monitoring being poorly correlated with AET measured on the first day. The above studies suggest that longer monitoring time may make AET and other indicators closer to real events and improve the accuracy of diagnosis.

### 4.7 Inspiration from research hotspots and emerging trends

The aforementioned research focal points and emerging trends will serve as valuable references for future research orientations in the subsequent areas. The first aspect involves the introduction of a new definition and diagnosis, where it is clinically essential to differentiate refractory GERD from refractory reflux-like symptoms and refractory GERD symptoms. Objective indicators (including emerging ones such as PSPW and MNBI) should be prioritized over subjective feelings as the key diagnostic criteria. This is beneficial in precisely identifying refractory GERD and facilitating a more objective and accurate assessment of the patient’s condition. Furthermore, an extended application of prolonged wireless pH monitoring would effectively reflect the most realistic reflux events and contribute to clinical diagnosis. This kind of precise diagnosis will undoubtedly become the prevailing trend in the future.

The second aspect involves the prognostication of therapeutic approaches grounded in innovative mechanisms. The release of pain mediators by immune cells can induce sensitization of peripheral nerves. Current research has demonstrated that there are numerous receptors upstream and downstream, which are associated with neuroimmunity, including neuropeptides, histamine, nociceptive mediators, NGF, and HIF-2α, may serve as crucial targets for the treatment of this disease and hold significant therapeutic potential. Therefore, the development of inhibitors such as NGF antagonists and HIF-2α inhibitors represents a promising direction for novel drug discovery. This is particularly advantageous for patients experiencing physiological acid regurgitation or non-acid regurgitation, with heartburn and retrosternal pain being the primary clinical manifestations.

The findings of various studies have demonstrated that 17-phenyl PGE2, acting as an EP1 agonist, exhibits a biphasic impact on esophageal mucosal inflammation. Specifically, it exerts a protective effect on the esophageal mucosa at lower doses (0.1 and 0.3 mg/kg), while displaying damaging effects at higher doses (1 mg/kg). Both of these effects are mediated through the activation of the EP1 receptor (Yamato et al., 2005; Takeuchi and Amagase, 2018). In addition, a randomized, single-blind, placebo-controlled, cross-over trial in recent years found that EP1 receptor may also be involved in the occurrence of TLESRs. An EP1 receptor antagonist, ONO-8539, has been shown to significantly reduce the frequency of TLESRs (Sawada et al., 2020).These findings suggest that targeting the EP1 receptor could be a promising therapeutic approach for patients with refractory GERD and further researches on drugs acting on EP1 receptor should be designed. The development of these novel drug types remains a highly active research field, playing a pivotal role in addressing the concerns of patients who are apprehensive about undergoing endoscopic or surgical interventions.

The third is that joint behavioral interventions may be beneficial. We hypothesized that developments in the field of psychogastroenterology may provide a new treatment for this disease until new breakthrough drugs are invented. Presently, an increasing number of studies are uncovering the significant role played by psychosocial factors in refractory GERD (Riehl and Chen, 2018; He et al., 2022) such as depression (Kimura et al., 2016), anxiety (Ribolsi et al., 2023) and sleep disorders (Kawara et al., 2017). The repeated and refractory discomfort experienced by patients often leads to feelings of disappointment and helplessness, which not only amplifies the perception of symptoms but also impacts medication adherence and responsiveness to medications such as PPIs.

For these patients, neuromodulators such as selective serotonin reuptake inhibitors (SSRIs) can serve as crucial adjuncts to stabilize mood and ameliorate symptoms. Fluoxetine demonstrated superiority over omeprazole in improving heartburn symptoms, particularly among patients without esophageal mucosa erosion and normal acid exposure (Ostovaneh et al., 2014). One study have shown that taking 20 mg of citalopram daily can improve symptoms in patients with hypersensitive esophagus by 61.5% compared to 33.3% with placebo (Viazis et al., 2012). Therefore, gastroenterologists should consider collaborating with psychological experts to conduct a thorough evaluation of patients with anxiety, depression, and other psychological disorders. In cases where these conditions coexist, combination therapy should be considered to enhance the efficacy of refractory GERD treatment. In addition, there is an increasing utilization of behavioral interventions targeting these underlying mechanisms, such as cognitive behavioral therapy (CBT), which advises clinicians to guide patients away from solely seeking all the symptoms relief. Encouraging the acceptance and tolerance of residual symptoms may help alleviate the negative emotions experienced by the patient (Riehl et al., 2015; Sawada et al., 2019). The intervention of diaphragmatic breathing training (DBT) has the potential to enhance therapeutic outcomes for GERD, while also contributing to improved sleep quality and overall life satisfaction. This improvement may be attributed to the mechanism of increasing the disparity between LES and stomach pressure to reduce postprandial reflux events (Halland et al., 2021; Zdrhova et al., 2023; Mosa et al., 2024). Moreover, Esophageal directed hypnotherapy has been shown to be an effective mode of treatment for regulating discomfort by adjusting the patient to a state of deep relaxation and concentration (Riehl et al., 2016).

### 4.8 Potential barriers and challenges

In fact, despite decades of extensive research, there are only two drugs, namely PPIs developed early on and P-CAB approved in 2016, which have profound impact and are widely recommended by consensus for the research field of GERD (whether RE, NERD or refractory GERD) (Talley and Zand, 2021). Currently, there are ongoing studies aimed at optimizing acid-suppressive therapy to enhance the management of refractory conditions. For example, from the perspective of pharmacokinetics and pharmacodynamics, they recommend refractory GERD patients to take acid inhibitors before meals as an important drug administration strategy because there is indirect evidence that this is more effective in alleviating symptoms (Zerbib et al., 2021a). Moreover, refractory GERD patients with persistent esophagitis or continuous esophageal acid exposure would derive greater benefits from stronger PPIs medications (Kinoshita et al., 2018). However, both drugs are essentially acid suppressants that act on the acid reflux mechanism by acting on the H+/K+-ATPase of gastric parietal cells. Given the intricate pathogenesis, an increasing number of researches have directed their attention towards factors other than acid reflux. For instance, the therapeutic effects of alginates are achieved by displacing the postprandial gastric acid pocket to alleviate distress in patients experiencing breakthrough symptoms (De et al., 2014; Leiman et al., 2017). Patients with persistent nocturnal acid breakthrough (impedance-pH monitoring suggested intragastric pH < 4 for more than one continuous hour overnight) can try to add Histamine type-2 receptor antagonists (H2RAs) at bedtime, but the supporting evidence is limited (Fackler et al., 2002; Mainie et al., 2008; Wang et al., 2009). The recent phase IIb study has confirmed that IW-3718, a gastric-retentive and extended-release formulation containing the bile acid sequestrant, effectively binds and sequesters bile acids enroute to the esophagus, providing significant relief for refractory heartburn and acid reflux symptoms (Vaezi et al., 2020). There are also Baclofen, a gamma-aminobutyric acid (GABA)-B receptor agonist and Prokinetics mentioned above. All of these medications have the characteristic of being only adjunctive or experimental to acid suppressant to attempt to alleviate the distress of patients with different clinical characteristics.

This poses the first significant challenge in the management of refractory GERD. Despite the development of various drugs targeting mechanisms beyond acid inhibition, their clinical application and evidence support remain limited, failing to provide definitive critical efficacy. Currently, there is an unmet need for new first-line drug options for refractory patients, as large-scale clinical studies lack evidence in this regard. Ongoing research focusing on novel mechanisms such as neuroimmunology and psychosomatic factors holds great promise.

The second challenge is that the pathogenesis of refractory GERD is not simple and homogenous, it is influenced by dozens of factors as mentioned above, which means that we cannot be sure which factor plays the most important role in this disease. In response, multi-targeted therapies and multidisciplinary collaborative treatment programs may be the way and the hope to overcome these barriers.

The third challenge lies in accurately identifying patients who truly have refractory GERD from a clinical perspective. A rigorous procedure should involve allowing patients to comply with an 8-week pre-treatment of PPIs, completing a questionnaire, and undergoing screening by specialists. Finally, objective indicators are utilized to confirm the suitability of conditions. It may even be necessary to employ prolonged wireless pH monitoring and conduct multiple high-resolution manometry tests. However, the current trend in clinical studies is to overlook this step and place greater emphasis on patients subjective outcomes. It is imperative for clinicians and researchers to allocate more attention to this aspect.

## 5 Conclusion

According to our bibliometrics insights, the following directions are expected to be developed in the future. Firstly, high-quality clinical studies should not overly rely on patients subjective perceptions but instead adhere strictly to diagnostic criteria and include only those patients who genuinely meet the requirements. This is crucial for minimizing misclassification bias in statistical findings. Secondly, the research in the field of neuroimmunology and psychopsychology is poised to become a pioneering area, with potential for significant advancements. The collaboration among diverse disciplines, including joint expertise from psychological professionals, can greatly facilitate the progress in this domain. Moreover, behavioral intervention is currently a prominent area of research and is anticipated to function as an alternative therapeutic approach until novel, stable medications targeting multiple mechanisms are developed. Finally, close cooperation between different disciplines and regions and keeping up with the frontiers of modern science and technology should be an effective way to promote the innovative development of research on refractory GERD.

It is worth emphasizing that, our study is the first bibliometric analysis of the global literature on refractory GERD, elucidating the research process and evolutionary trends in this field from 2000 to 2023. This work visually demonstrates the development status, cooperative relationship, research focus, and possible hotspots of this disease. Currently, research in this area focuses on standardized diagnosis and management, and in-depth exploration of novel mechanisms such as neuroimmune interaction. Meanwhile, efforts should also be made to design simpler, more accurate, and more stable monitoring methods and seek new treatment options based on different mechanisms, including the development of innovative drugs and procedures. In conclusion, it is hoped that this research will provide valuable insights for researchers, enabling them to quickly and comprehensively understand the historical background, current status, and future directions of refractory GERD.

### 5.1 Limitations

This study has limitations. Firstly, due to the constraints of bibliometric tools, we exclusively relied on the more authoritative and comprehensive database-Web of Science for data retrieval. Secondly, although our retrieval method was designed to be relatively comprehensive, it inevitably failed to capture certain articles. Additionally, subjective and professional experience limitations may have influenced the data-cleaning process. These above issues might have impacted the accuracy of our retrieved data.

## Data Availability

The original contributions presented in the study are included in the article/Supplementary Material, further inquiries can be directed to the corresponding author.

## References

[B1] Abe Y. Koike T. Saito M. Okata T. Nakagawa K. Hatta W. (2021). The ameliorating effect of switching to vonoprazan: a novel potassium-competitive acid blocker in patients with proton pump inhibitor refractory non-erosive reflux disease. Digestion 102 (3), 480–488. 10.1159/000506152 32062650

[B2] Allison P. R. (1951). Reflux esophagitis, sliding hiatal hernia, and the anatomy of repair. Surg. Gynecol. Obstet. 92 (4), 419–431. 14835197

[B3] Argüero J. Sifrim D. (2024). Pathophysiology of gastro-oesophageal reflux disease: implications for diagnosis and management. Nat. Rev. Gastroenterol. Hepatol. 4, 282–293. Published online January. 10.1038/s41575-023-00883-z 38177402

[B4] Armstrong D. Hungin A. P. Kahrilas P. J. Sifrim D. Sinclair P. Vaezi M. F. (2022). Knowledge gaps in the management of refractory reflux-like symptoms: healthcare provider survey. Neurogastroenterol. Motil. 34 (10), e14387. 10.1111/nmo.14387 35502888 PMC9787909

[B5] Ashida K. Sakurai Y. Hori T. Kudou K. Nishimura A. Hiramatsu N. (2016). Randomised clinical trial: vonoprazan, a novel potassium-competitive acid blocker, vs. lansoprazole for the healing of erosive oesophagitis. Aliment. Pharmacol. Ther. 43 (2), 240–251. 10.1111/apt.13461 26559637 PMC4738414

[B6] Aziz Q. Fass R. Gyawali C. P. Miwa H. Pandolfino J. E. Zerbib F. (2016). Esophageal disorders. Gastroenterology 150, 1368–1379. 10.1053/j.gastro.2016.02.012 27144625

[B7] Charbel S. Khandwala F. Vaezi M. F. (2005). The role of esophageal pH monitoring in symptomatic patients on PPI therapy. Am. J. Gastroenterol. 100 (2), 283–289. 10.1111/j.1572-0241.2005.41210.x 15667483

[B8] Chen C. (2006). CiteSpace II: detecting and visualizing emerging trends and transient patterns in scientific literature. J. Am. Soc. Inf. Sci. Technol. 57 (3), 359–377. 10.1002/asi.20317

[B9] Chen C. Hu Z. Liu S. Tseng H. (2012). Emerging trends in regenerative medicine: a scientometric analysis in CiteSpace. Expert Opin. Biol. Ther. 12 (5), 593–608. 10.1517/14712598.2012.674507 22443895

[B10] Chey W. D. Mody R. R. Izat E. (2010). Patient and physician satisfaction with proton pump inhibitors (PPIs): are there opportunities for improvement? Dig. Dis. Sci. 55 (12), 3415–3422. 10.1007/s10620-010-1209-2 20397047

[B11] Code C. F. Fyke F. E. Schlegel J. F. (1956). The gastroesophageal sphincter in healthy human beings. Gastroenterologia 86 (3), 135–150. 10.1159/000200544 13384582

[B12] Dallemagne B. Weerts J. M. Jehaes C. Markiewicz S. Lombard R. (1991). Laparoscopic Nissen fundoplication: preliminary report. Surg. Laparosc. Endosc. 1 (3), 138–143. 1669393

[B13] De R. A. Roman S. Chen J. Pandolfino J. E. Kahrilas P. J. (2014). Gaviscon Double Action Liquid (antacid and alginate) is more effective than antacid in controlling post-prandial oesophageal acid exposure in GERD patients: a double-blind crossover study. Aliment. Pharmacol. Ther. 40 (5), 531–537. 10.1111/apt.12857 25041141 PMC4343538

[B14] Delshad S. D. Almario C. V. Chey W. D. Spiegel B. M. R. (2020). Prevalence of gastroesophageal reflux disease and proton pump inhibitor-refractory symptoms. Gastroenterology 158 (5), 1250–1261. 10.1053/j.gastro.2019.12.014 31866243 PMC7103516

[B15] Dent J. Dodds W. J. Friedman R. H. Sekiguchi T. Hogan W. J. Arndorfer R. C. (1980). Mechanism of gastroesophageal reflux in recumbent asymptomatic human subjects. J. Clin. Investig. 65 (2), 256–267. 10.1172/JCI109667 7356677 PMC371362

[B16] Dent J. Kahrilas P. J. Hatlebakk J. Vakil N. Denison H. Franzén S. (2008). A randomized, comparative trial of a potassium-competitive acid blocker (AZD0865) and esomeprazole for the treatment of patients with nonerosive reflux disease. Am. J. Gastroenterol. 103 (1), 20–26. 10.1111/j.1572-0241.2007.01544.x 18184117

[B17] Dobashi A. Li D. K. Mavrogenis G. Visrodia K. H. Bazerbachi F. (2022). Endoscopic management of esophageal cancer. Thorac. Surg. Clin. 32 (4), 479–495. 10.1016/j.thorsurg.2022.07.005 36266035

[B18] Dothel G. Barbaro M. R. Boudin H. Vasina V. Cremon C. Gargano L. (2015). Nerve fiber outgrowth is increased in the intestinal mucosa of patients with irritable bowel syndrome. Gastroenterology 148 (5), 1002–1011. 10.1053/j.gastro.2015.01.042 25655556

[B19] Dotsika F. Watkins A. (2017). Identifying potentially disruptive trends by means of keyword network analysis. Technol. Forecast Soc. Change 119, 114–127. 10.1016/j.techfore.2017.03.020

[B20] Dunbar K. B. Agoston A. T. Odze R. D. Huo X. Pham T. H. Cipher D. J. (2016). Association of acute gastroesophageal reflux disease with esophageal histologic changes. JAMA 315 (19), 2104–2112. 10.1001/jama.2016.5657 27187303 PMC5030713

[B21] El-Serag H. Becher A. Jones R. (2010a). Systematic review: persistent reflux symptoms on proton pump inhibitor therapy in primary care and community studies. Aliment. Pharmacol. Ther. 32 (6), 720–737. 10.1111/j.1365-2036.2010.04406.x 20662774

[B22] El-Serag H. Becher A. Jones R. (2010b). Systematic review: persistent reflux symptoms on proton pump inhibitor therapy in primary care and community studies. Aliment. Pharmacol. Ther. 32 (6), 720–737. 10.1111/j.1365-2036.2010.04406.x 20662774

[B23] El-Serag H. B. (2007). Time trends of gastroesophageal reflux disease: a systematic review. Clin. Gastroenterol. Hepatol. Off. Clin. Pract. J. Am. Gastroenterol. Assoc. 5 (1), 17–26. 10.1016/j.cgh.2006.09.016 17142109

[B24] Eskander M. A. Ruparel S. Green D. P. Chen P. B. Por E. D. Jeske N. A. (2015). Persistent nociception triggered by nerve growth factor (NGF) is mediated by TRPV1 and oxidative mechanisms. J. Neurosci. Off. J. Soc. Neurosci. 35 (22), 8593–8603. 10.1523/JNEUROSCI.3993-14.2015 PMC4452557 26041925

[B25] Fackler W. K. Ours T. M. Vaezi M. F. Richter J. E. (2002). Long-term effect of H2RA therapy on nocturnal gastric acid breakthrough. Gastroenterology 122 (3), 625–632. 10.1053/gast.2002.31876 11874994

[B26] Fass R. Murthy U. Hayden C. W. Malagon I. B. Pulliam G. Wendel C. (2000). Omeprazole 40 mg once a day is equally effective as lansoprazole 30 mg twice a day in symptom control of patients with gastro-oesophageal reflux disease (GERD) who are resistant to conventional-dose lansoprazole therapy-a prospective, randomized, multi-centre study. Aliment. Pharmacol. Ther. 14 (12), 1595–1603. 10.1046/j.1365-2036.2000.00882.x 11121907

[B27] Fass R. Sifrim D. (2009a). Management of heartburn not responding to proton pump inhibitors. Gut 58 (2), 295–309. 10.1136/gut.2007.145581 19136523

[B28] Fass R. Sifrim D. (2009b). Management of heartburn not responding to proton pump inhibitors. Gut 58 (2), 295–309. 10.1136/gut.2007.145581 19136523

[B29] Fock K. M. Talley N. Goh K. L. Sugano K. Katelaris P. Holtmann G. (2016). Asia-Pacific consensus on the management of gastro-oesophageal reflux disease: an update focusing on refractory reflux disease and Barrett’s oesophagus. Gut 65 (9), 1402–1415. 10.1136/gutjnl-2016-311715 27261337

[B30] Frazzoni M. Conigliaro R. Melotti G. (2011a). Reflux parameters as modified by laparoscopic fundoplication in 40 patients with heartburn/regurgitation persisting despite PPI therapy: a study using impedance-pH monitoring. Dig. Dis. Sci. 56 (4), 1099–1106. 10.1007/s10620-010-1381-4 20737211

[B31] Frazzoni M. Conigliaro R. Melotti G. (2011b). Weakly acidic refluxes have a major role in the pathogenesis of proton pump inhibitor-resistant reflux oesophagitis. Aliment. Pharmacol. Ther. 33 (5), 601–606. 10.1111/j.1365-2036.2010.04550.x 21198705

[B32] Frazzoni M. de Bortoli N. Frazzoni L. Tolone S. Furnari M. Martinucci I. (2017). The added diagnostic value of postreflux swallow-induced peristaltic wave index and nocturnal baseline impedance in refractory reflux disease studied with on-therapy impedance-pH monitoring. Neurogastroenterol. Motil. 29 (3). 10.1111/nmo.12947 27620303

[B33] Frazzoni M. Frazzoni L. Ribolsi M. Russo S. Conigliaro R. De Bortoli N. (2023). On-therapy impedance-pH monitoring can efficiently characterize PPI-refractory GERD and support treatment escalation. Neurogastroenterol. Motil. 35 (5), e14547. 10.1111/nmo.14547 36780512

[B34] Fujishiro M. Matsumoto T. (2022). History of endoscopes: contribution of the Japan gastroenterological endoscopy society. Dig. Endosc. Off. J. Jpn. Gastroenterol. Endosc. Soc. 34 (Suppl. 2), 13–14. 10.1111/den.14111 34486761

[B35] Garnock-Jones K. P. (2015). Vonoprazan: first global approval. Drugs 75 (4), 439–443. 10.1007/s40265-015-0368-z 25744862

[B36] Gawron A. J. Rothe J. Fought A. J. Fareeduddin A. Toto E. Boris L. (2012). Many patients continue using proton pump inhibitors after negative results from tests for reflux disease. Clin. Gastroenterol. Hepatol. Off. Clin. Pract. J. Am. Gastroenterol. Assoc. 10 (6), 620–625. 10.1016/j.cgh.2012.02.012 PMC3547497 22366177

[B37] Gerson L. B. Bonafede M. Princic N. Gregory C. Farr A. Balu S. (2011). Development of a refractory gastro-oesophageal reflux score using an administrative claims database. Aliment. Pharmacol. Ther. 34 (5), 555–567. 10.1111/j.1365-2036.2011.04755.x 21714794

[B38] Grubic A. D. Crookes P. F. (2021). Evolution of esophageal motility testing: from kronecker to clouse. Foregut J. Am. Foregut Soc. 1 (3), 197–206. 10.1177/26345161211044151

[B39] Guadagnoli L. Yadlapati R. Taft T. Pandolfino J. E. Tye M. Keefer L. (2021). Esophageal hypervigilance is prevalent across gastroesophageal reflux disease presentations. Neurogastroenterol. Motil. 33 (8), e14081. 10.1111/nmo.14081 33432708 PMC8272741

[B40] Guarino M. P. L. Cheng L. Ma J. Harnett K. Biancani P. Altomare A. (2010). Increased TRPV1 gene expression in esophageal mucosa of patients with non-erosive and erosive reflux disease. Neurogastroenterol. Motil. 22 (7), 746–751. 10.1111/j.1365-2982.2010.01514.x 20456759

[B41] Gupta K. Harvima I. T. (2018). Mast cell-neural interactions contribute to pain and itch. Immunol. Rev. 282 (1), 168–187. 10.1111/imr.12622 29431216 PMC5812374

[B42] Gyawali C. P. Kahrilas P. J. Savarino E. Zerbib F. Mion F. Smout A. J. P. M. (2018a). Modern diagnosis of GERD: the Lyon consensus. Gut 67 (7), 1351–1362. 10.1136/gutjnl-2017-314722 29437910 PMC6031267

[B43] Gyawali C. P. Kahrilas P. J. Savarino E. Zerbib F. Mion F. Smout A. J. P. M. (2018b). Modern diagnosis of GERD: the Lyon consensus. Gut 67 (7), 1351–1362. 10.1136/gutjnl-2017-314722 29437910 PMC6031267

[B44] Gyawali C. P. Tutuian R. Zerbib F. Rogers B. D. Frazzoni M. Roman S. (2021). Value of pH impedance monitoring while on twice-daily proton pump inhibitor therapy to identify need for escalation of reflux management. Gastroenterology 161 (5), 1412–1422. 10.1053/j.gastro.2021.07.004 34270955

[B45] Gyawali C. P. Yadlapati R. Fass R. Katzka D. Pandolfino J. Savarino E. (2023). Updates to the modern diagnosis of GERD: Lyon consensus 2.0. Gut 73, 361–371. 10.1136/gutjnl-2023-330616 PMC10846564 37734911

[B46] Halland M. Bharucha A. E. Crowell M. D. Ravi K. Katzka D. A. (2021). Effects of diaphragmatic breathing on the pathophysiology and treatment of upright gastroesophageal reflux: a randomized controlled trial. Am. J. Gastroenterol. 116 (1), 86–94. 10.14309/ajg.0000000000000913 33009052

[B47] Hasak S. Yadlapati R. Altayar O. Sweis R. Tucker E. Knowles K. (2020). Prolonged wireless pH monitoring in patients with persistent reflux symptoms despite proton pump inhibitor therapy. Clin. Gastroenterol. Hepatol. Off. Clin. Pract. J. Am. Gastroenterol. Assoc. 18 (13), 2912–2919. 10.1016/j.cgh.2020.01.031 PMC7392797 32007543

[B48] He M. Wang Q. Yao D. Li J. Bai G. (2022). Association between psychosocial disorders and gastroesophageal reflux disease: a systematic review and meta-analysis. J. Neurogastroenterol. Motil. 28 (2), 212–221. 10.5056/jnm21044 35362447 PMC8978133

[B49] Hemmink G. J. M. Bredenoord A. J. Weusten BLAM Monkelbaan J. F. Timmer R. Smout AJPM (2008a). Esophageal pH-impedance monitoring in patients with therapy-resistant reflux symptoms: “on” or “off” proton pump inhibitor? Am. J. Gastroenterol. 103 (10), 2446–2453. 10.1111/j.1572-0241.2008.02033.x 18684197

[B50] Hemmink G. J. M. Bredenoord A. J. Weusten BLAM Monkelbaan J. F. Timmer R. Smout AJPM (2008b). Esophageal pH-impedance monitoring in patients with therapy-resistant reflux symptoms: “on” or “off” proton pump inhibitor? Am. J. Gastroenterol. 103 (10), 2446–2453. 10.1111/j.1572-0241.2008.02033.x 18684197

[B51] Herregods T. V. K. Troelstra M. Weijenborg P. W. Bredenoord A. J. Smout AJPM (2015). Patients with refractory reflux symptoms often do not have GERD. Neurogastroenterol. Motil. 27 (9), 1267–1273. 10.1111/nmo.12620 26088946

[B52] Hershcovici T. Mashimo H. Fass R. (2011). The lower esophageal sphincter. Neurogastroenterol. Motil. 23 (9), 819–830. 10.1111/j.1365-2982.2011.01738.x 21711416

[B53] Hoshino S. Kawami N. Takenouchi N. Umezawa M. Hanada Y. Hoshikawa Y. (2017a). Efficacy of vonoprazan for proton pump inhibitor-resistant reflux esophagitis. Digestion 95 (2), 156–161. 10.1159/000456072 28190016

[B54] Hoshino S. Kawami N. Takenouchi N. Umezawa M. Hanada Y. Hoshikawa Y. (2017b). Efficacy of vonoprazan for proton pump inhibitor-resistant reflux esophagitis. Digestion 95 (2), 156–161. 10.1159/000456072 28190016

[B55] Howden C. W. Manuel M. Taylor D. Jariwala-Parikh K. Tkacz J. (2021). Estimate of refractory reflux disease in the United States: economic burden and associated clinical characteristics. J. Clin. Gastroenterol. 55 (10), 842–850. 10.1097/MCG.0000000000001518 33780218

[B56] Huo X. Agoston A. T. Dunbar K. B. Cipher D. J. Zhang X. Yu C. (2017). Hypoxia-inducible factor-2α plays a role in mediating oesophagitis in GORD. Gut 66 (9), 1542–1554. 10.1136/gutjnl-2016-312595 27694141 PMC5464991

[B57] Ishimura N. Mori M. Mikami H. Shimura S. Uno G. Aimi M. (2015). Effects of acotiamide on esophageal motor function and gastroesophageal reflux in healthy volunteers. BMC Gastroenterol. 15, 117. 10.1186/s12876-015-0346-7 26362795 PMC4567836

[B58] Iwakiri K. Kinoshita Y. Habu Y. Oshima T. Manabe N. Fujiwara Y. (2016). Evidence-based clinical practice guidelines for gastroesophageal reflux disease 2015. J. Gastroenterol. 51 (8), 751–767. 10.1007/s00535-016-1227-8 27325300

[B59] Jung D. H. Huh C. W. Lee S. K. Park J. C. Shin S. K. Lee Y. C. (2021). A systematic review and meta-analysis of randomized control trials: combination treatment with proton pump inhibitor plus prokinetic for gastroesophageal reflux disease. J. Neurogastroenterol. Motil. 27 (2), 165–175. 10.5056/jnm20161 33795539 PMC8026378

[B60] Kagami T. Sahara S. Ichikawa H. Uotani T. Yamade M. Sugimoto M. (2016). Potent acid inhibition by vonoprazan in comparison with esomeprazole, with reference to CYP2C19 genotype. Aliment. Pharmacol. Ther. 43 (10), 1048–1059. 10.1111/apt.13588 26991399

[B61] Kahrilas P. J. (2010). Esophageal motor disorders in terms of high-resolution esophageal pressure topography: what has changed? Am. J. Gastroenterol. 105 (5), 981–987. 10.1038/ajg.2010.43 20179690 PMC2888528

[B62] Kahrilas P. J. Bredenoord A. J. Fox M. Gyawali C. P. Roman S. Smout A. J. P. M. (2015). The Chicago Classification of esophageal motility disorders, v3.0. Neurogastroenterol. Motil. 27 (2), 160–174. 10.1111/nmo.12477 25469569 PMC4308501

[B63] Kahrilas P. J. Ghosh S. K. Pandolfino J. E. (2008b). Esophageal motility disorders in terms of pressure topography: the Chicago Classification. J. Clin. Gastroenterol. 42 (5), 627–635. 10.1097/MCG.0b013e31815ea291 18364587 PMC2895002

[B64] Kahrilas P. J. Howden C. W. Wernersson B. Denison H. Nuevo J. Gisbert J. P. (2013). Impact of persistent, frequent regurgitation on quality of life in heartburn responders treated with acid suppression: a multinational primary care study. Aliment. Pharmacol. Ther. 37 (10), 1005–1010. 10.1111/apt.12298 23557078

[B65] Kahrilas P. J. Jonsson A. Denison H. Wernersson B. Hughes N. Howden C. W. (2012). Regurgitation is less responsive to acid suppression than heartburn in patients with gastroesophageal reflux disease. Clin. Gastroenterol. Hepatol. Off. Clin. Pract. J. Am. Gastroenterol. Assoc. 10 (6), 612–619. 10.1016/j.cgh.2012.01.022 22343515

[B66] Kahrilas P. J. Jonsson A. Denison H. Wernersson B. Hughes N. Howden C. W. (2014). Impact of regurgitation on health-related quality of life in gastro-oesophageal reflux disease before and after short-term potent acid suppression therapy. Gut 63 (5), 720–726. 10.1136/gutjnl-2013-304883 23831734 PMC4124076

[B67] Kahrilas P. J. Shaheen N. J. Vaezi M. F. (2008a). American Gastroenterological Association Institute technical review on the management of gastroesophageal reflux disease. Gastroenterology 135 (4), 1392–1413.e14135. 10.1053/j.gastro.2008.08.044 18801365

[B68] Kalapala R. Singla N. Reddy D. N. (2022). Endoscopic management of gastroesophageal reflux disease: panacea for proton pump inhibitors dependent/refractory patients. Dig. Endosc. Off. J. Jpn. Gastroenterol. Endosc. Soc. 34 (4), 687–699. 10.1111/den.14169 34651353

[B69] Katz P. O. Dunbar K. B. Schnoll-Sussman F. H. Greer K. B. Yadlapati R. Spechler S. J. (2022). ACG clinical guideline for the diagnosis and management of gastroesophageal reflux disease. Am. J. Gastroenterol. 117 (1), 27–56. 10.14309/ajg.0000000000001538 34807007 PMC8754510

[B70] Katz P. O. Gerson L. B. Vela M. F. (2013a). Guidelines for the diagnosis and management of gastroesophageal reflux disease. Am. J. Gastroenterol. 108 (3), 308–328. quiz 329. 10.1038/ajg.2012.444 23419381

[B71] Katz P. O. Gerson L. B. Vela M. F. (2013b). Guidelines for the diagnosis and management of gastroesophageal reflux disease. Am. J. Gastroenterol. 108 (3), 308–328. quiz 329. 10.1038/ajg.2012.444 23419381

[B72] Katz P. O. Gerson L. B. Vela M. F. (2013c). Guidelines for the diagnosis and management of gastroesophageal reflux disease. Am. J. Gastroenterol. 108 (3), 308–328. quiz 329. 10.1038/ajg.2012.444 23419381

[B73] Kawami N. Hoshino S. Hoshikawa Y. Takenouchi N. Umezawa M. Hanada Y. (2018). Pathogenesis of potassium-competitive acid blocker-resistant non-erosive reflux disease. Digestion 98 (3), 194–200. 10.1159/000488530 29870976

[B74] Kawara F. Fujita T. Morita Y. Uda A. Masuda A. Saito M. (2017). Factors associated with residual gastroesophageal reflux disease symptoms in patients receiving proton pump inhibitor maintenance therapy. World J. Gastroenterol. 23 (11), 2060–2067. 10.3748/wjg.v23.i11.2060 28373773 PMC5360648

[B75] Kent C. N. Park C. Lindsley C. W. (2020). Classics in chemical neuroscience: baclofen. ACS Chem. Neurosci. 11 (12), 1740–1755. 10.1021/acschemneuro.0c00254 32436697

[B76] Kim S. E. Kim N. Oh S. Kim H. M. Park M. I. Lee D. H. (2015). Predictive factors of response to proton pump inhibitors in Korean patients with gastroesophageal reflux disease. J. Neurogastroenterol. Motil. 21 (1), 69–77. 10.5056/jnm14078 25537676 PMC4288103

[B77] Kimura Y. Kamiya T. Senoo K. Tsuchida K. Hirano A. Kojima H. (2016). Persistent reflux symptoms cause anxiety, depression, and mental health and sleep disorders in gastroesophageal reflux disease patients. J. Clin. Biochem. Nutr. 59 (1), 71–77. 10.3164/jcbn.16-9 27499583 PMC4933696

[B78] Kinoshita Y. Kato M. Fujishiro M. Masuyama H. Nakata R. Abe H. (2018). Efficacy and safety of twice-daily rabeprazole maintenance therapy for patients with reflux esophagitis refractory to standard once-daily proton pump inhibitor: the Japan-based EXTEND study. J. Gastroenterol. 53 (7), 834–844. 10.1007/s00535-017-1417-z 29188387 PMC6006226

[B79] Klinkenberg-Knol E. C. Nelis F. Dent J. Snel P. Mitchell B. Prichard P. (2000). Long-term omeprazole treatment in resistant gastroesophageal reflux disease: efficacy, safety, and influence on gastric mucosa. Gastroenterology 118 (4), 661–669. 10.1016/s0016-5085(00)70135-1 10734017

[B80] Koek G. H. Sifrim D. Lerut T. Janssens J. Tack J. (2003). Effect of the GABA(B) agonist baclofen in patients with symptoms and duodeno-gastro-oesophageal reflux refractory to proton pump inhibitors. Gut 52 (10), 1397–1402. 10.1136/gut.52.10.1397 12970129 PMC1773817

[B81] Kohata Y. Fujiwara Y. Machida H. Okazaki H. Yamagami H. Tanigawa T. (2012). Pathogenesis of proton-pump inhibitor-refractory non-erosive reflux disease according to multichannel intraluminal impedance-pH monitoring. J. Gastroenterol. Hepatol. 27 (Suppl. 3), 58–62. 10.1111/j.1440-1746.2012.07074.x 22486873

[B82] Leech T. Peiris M. (2024). Mucosal neuroimmune mechanisms in gastro-oesophageal reflux disease (GORD) pathogenesis. J. Gastroenterol. 59, 165–178. 10.1007/s00535-023-02065-9 38221552 PMC10904498

[B83] Leiman D. A. Riff B. P. Morgan S. Metz D. C. Falk G. W. French B. (2017). Alginate therapy is effective treatment for GERD symptoms: a systematic review and meta-analysis. Dis. Esophagus Off. J. Int. Soc. Dis. Esophagus 30 (5), 1–9. 10.1093/dote/dow020 PMC6036656 28375448

[B84] Leite L. P. Johnston B. T. Barrett J. Castell J. A. Castell D. O. (1997). Ineffective esophageal motility (IEM): the primary finding in patients with nonspecific esophageal motility disorder. Dig. Dis. Sci. 42 (9), 1859–1865. 10.1023/a:1018802908358 9331148

[B85] Li K. Rollins J. Yan E. (2018). Web of Science use in published research and review papers 1997-2017: a selective, dynamic, cross-domain, content-based analysis. Scientometrics 115 (1), 1–20. 10.1007/s11192-017-2622-5 29527070 PMC5838136

[B86] Mainie I. Tutuian R. Castell D. O. (2008). Addition of a H2 receptor antagonist to PPI improves acid control and decreases nocturnal acid breakthrough. J. Clin. Gastroenterol. 42 (6), 676–679. 10.1097/MCG.0b013e31814a4e5c 18496394

[B87] Mainie I. Tutuian R. Shay S. Vela M. Zhang X. Sifrim D. (2006). Acid and non-acid reflux in patients with persistent symptoms despite acid suppressive therapy: a multicentre study using combined ambulatory impedance-pH monitoring. Gut 55 (10), 1398–1402. 10.1136/gut.2005.087668 16556669 PMC1856433

[B88] Martinez S. D. Malagon I. B. Garewal H. S. Cui H. Fass R. (2003). Non-erosive reflux disease (NERD)--acid reflux and symptom patterns. Aliment. Pharmacol. Ther. 17 (4), 537–545. 10.1046/j.1365-2036.2003.01423.x 12622762

[B89] McCarty T. R. Itidiare M. Njei B. Rustagi T. (2018). Efficacy of transoral incisionless fundoplication for refractory gastroesophageal reflux disease: a systematic review and meta-analysis. Endoscopy 50 (7), 708–725. 10.1055/a-0576-6589 29625507

[B90] Miwa H. Igarashi A. Teng L. Uda A. Deguchi H. Tango T. (2019). Systematic review with network meta-analysis: indirect comparison of the efficacy of vonoprazan and proton-pump inhibitors for maintenance treatment of gastroesophageal reflux disease. J. Gastroenterol. 54 (8), 718–729. 10.1007/s00535-019-01572-y 30919071 PMC6647489

[B91] Mosa H. E. S. El-Bready H. G. El-Sol AESH Bayomy H. E. Taman R. O. Shehata H. S. (2024). Efficacy of abdominal breathing on sleep and quality of life among patients with non-erosive gastroesophageal reflux. J. Public Health Res. 13 (1), 22799036241231788. 10.1177/22799036241231788 38370147 PMC10874155

[B92] Nirwan J. S. Hasan S. S. Babar Z. U. D. Conway B. R. Ghori M. U. (2020). Global prevalence and risk factors of gastro-oesophageal reflux disease (GORD): systematic review with meta-analysis. Sci. Rep. 10 (1), 5814. 10.1038/s41598-020-62795-1 32242117 PMC7118109

[B93] Nissen R. (1956). A simple operation for control of reflux esophagitis. Schweiz Med. Wochenschr 86 (Suppl. 20), 590–592. 13337262

[B94] Noar M. Squires P. Noar E. Lee M. (2014). Long-term maintenance effect of radiofrequency energy delivery for refractory GERD: a decade later. Surg. Endosc. 28 (8), 2323–2333. 10.1007/s00464-014-3461-6 24562599

[B95] Ochiai Y. Iizuka T. Hoshihara Y. Suzuki Y. Hayasaka J. Nomura K. (2021). Efficacy of vonoprazan for refractory reflux esophagitis after esophagectomy. Dig. Dis. Basel Switz. 39 (6), 569–576. 10.1159/000515146 PMC8686710 33567428

[B96] Olsen A. M. Schlegel J. F. Creamer B. Ellis F. H. (1957). Esophageal motility in achalasia (cardiospasm) after treatment. J. Thorac. Surg. 34 (5), 615–623. 10.1016/s0096-5588(20)30310-x 13476469

[B97] Ostovaneh M. R. Saeidi B. Hajifathalian K. Farrokhi-Khajeh-Pasha Y. Fotouhi A. Mirbagheri S. S. (2014). Comparing omeprazole with fluoxetine for treatment of patients with heartburn and normal endoscopy who failed once daily proton pump inhibitors: double-blind placebo-controlled trial. Neurogastroenterol. Motil. Off. J. Eur. Gastrointest. Motil. Soc. 26 (5), 670–678. 10.1111/nmo.12313 24533896

[B98] Patel S. H. Smith B. Polak R. Pomeranz M. Patel P. V. Englehardt R. (2022). Laparoscopic magnetic sphincter augmentation device placement for patients with medically-refractory gastroesophageal reflux after sleeve gastrectomy. Surg. Endosc. 36 (11), 8255–8260. 10.1007/s00464-022-09261-3 35474390

[B99] Pathirana A. Poston G. J. (2001). Lessons from Japan--endoscopic management of early gastric and oesophageal cancer. Eur. J. Surg. Oncol. J. Eur. Soc. Surg. Oncol. Br. Assoc. Surg. Oncol. 27 (1), 9–16. 10.1053/ejso.2000.1041 11237485

[B100] Pauwels A. Raymenants K. Geeraerts A. Boecxstaens V. Masuy I. Broers C. (2022). Clinical trial: a controlled trial of baclofen add-on therapy in PPI-refractory gastro-oesophageal reflux symptoms. Aliment. Pharmacol. Ther. 56 (2), 231–239. 10.1111/apt.17068 35665521

[B101] Penagini R. Sweis R. Mauro A. Domingues G. Vales A. Sifrim D. (2015a). Inconsistency in the diagnosis of functional heartburn: usefulness of prolonged wireless pH monitoring in patients with proton pump inhibitor refractory gastroesophageal reflux disease. J. Neurogastroenterol. Motil. 21 (2), 265–272. 10.5056/jnm14075 25843078 PMC4398246

[B102] Penagini R. Sweis R. Mauro A. Domingues G. Vales A. Sifrim D. (2015b). Inconsistency in the diagnosis of functional heartburn: usefulness of prolonged wireless pH monitoring in patients with proton pump inhibitor refractory gastroesophageal reflux disease. J. Neurogastroenterol. Motil. 21 (2), 265–272. 10.5056/jnm14075 25843078 PMC4398246

[B103] Raymenants K. Vandenbergh J. Tack J. (2022). Letter: should we have to include baclofen in the GORD therapy armamentarium? authors’ reply. Aliment. Pharmacol. Ther. 56 (8), 1308–1309. 10.1111/apt.17214 36168259

[B104] Ribolsi M. Marchetti L. Blasi V. Cicala M. (2023). Anxiety correlates with excessive air swallowing and PPI refractoriness in patients with concomitant symptoms of GERD and functional dyspepsia. Neurogastroenterol. Motil. 35 (7), e14550. 10.1111/nmo.14550 36786093

[B105] Richter J. E. (2006). The patient with refractory gastroesophageal reflux disease. Dis. Esophagus 19 (6), 443–447. 10.1111/j.1442-2050.2006.00619.x 17069586

[B106] Riehl M. E. Chen J. W. (2018). The proton pump inhibitor nonresponder: a behavioral approach to improvement and wellness. Curr. Gastroenterol. Rep. 20 (7), 34. 10.1007/s11894-018-0641-x 29886565

[B107] Riehl M. E. Kinsinger S. Kahrilas P. J. Pandolfino J. E. Keefer L. (2015). Role of a health psychologist in the management of functional esophageal complaints. Dis. Esophagus Off. J. Int. Soc. Dis. Esophagus 28 (5), 428–436. 10.1111/dote.12219 PMC4703073 26174953

[B108] Riehl M. E. Pandolfino J. E. Palsson O. S. Keefer L. (2016). Feasibility and acceptability of esophageal-directed hypnotherapy for functional heartburn. Dis. Esophagus Off. J. Int. Soc. Dis. Esophagus. 29 (5), 490–496. 10.1111/dote.12353 PMC4589470 25824436

[B109] Roark R. Sydor M. Chatila A. T. Umar S. Guerra R. D. L. Bilal M. (2020). Management of gastroesophageal reflux disease. Dis--Mon DM 66 (1), 100849. 10.1016/j.disamonth.2019.02.002 30798984

[B110] Rohof W. O. Bennink R. J. de Jonge H. Boeckxstaens G. E. (2014). Increased proximal reflux in a hypersensitive esophagus might explain symptoms resistant to proton pump inhibitors in patients with gastroesophageal reflux disease. Clin. Gastroenterol. Hepatol. Off. Clin. Pract. J. Am. Gastroenterol. Assoc. 12 (10), 1647–1655. 10.1016/j.cgh.2013.10.026 24184737

[B111] Rosa A. C. Fantozzi R. (2013). The role of histamine in neurogenic inflammation. Br. J. Pharmacol. 170 (1), 38–45. 10.1111/bph.12266 23734637 PMC3764847

[B112] Sakurai Y. Mori Y. Okamoto H. Nishimura A. Komura E. Araki T. (2015). Acid-inhibitory effects of vonoprazan 20 mg compared with esomeprazole 20 mg or rabeprazole 10 mg in healthy adult male subjects--a randomised open-label cross-over study. Aliment. Pharmacol. Ther. 42 (6), 719–730. 10.1111/apt.13325 26193978

[B113] Sawada A. Anastasi N. Green A. Glasinovic E. Wynter E. Albusoda A. (2019). Management of supragastric belching with cognitive behavioural therapy: factors determining success and follow-up outcomes at 6-12 months post-therapy. Aliment. Pharmacol. Ther. 50 (5), 530–537. 10.1111/apt.15417 31339173

[B114] Sawada A. Hashimoto A. Uemura R. Yamagami H. Tanigawa T. Watanabe T. (2020). Effect of EP1 receptor antagonist on transient lower esophageal sphincter relaxations in humans. Digestion 101 (3), 270–278. 10.1159/000499333 30897584

[B115] Scarpellini E. Ang D. Pauwels A. De Santis A. Vanuytsel T. Tack J. (2016). Management of refractory typical GERD symptoms. Nat. Rev. Gastroenterol. Hepatol. 13 (5), 281–294. 10.1038/nrgastro.2016.50 27075264

[B116] Sharma N. Agrawal A. Freeman J. Vela M. F. Castell D. (2008). An analysis of persistent symptoms in acid-suppressed patients undergoing impedance-pH monitoring. Clin. Gastroenterol. Hepatol. Off. Clin. Pract. J. Am. Gastroenterol. Assoc. 6 (5), 521–524. 10.1016/j.cgh.2008.01.006 18356117

[B117] Sifrim D. Castell D. Dent J. Kahrilas P. J. (2004). Gastro-oesophageal reflux monitoring: review and consensus report on detection and definitions of acid, non-acid, and gas reflux. Gut 53 (7), 1024–1031. 10.1136/gut.2003.033290 15194656 PMC1774114

[B118] Sifrim D. Zerbib F. (2012a). Diagnosis and management of patients with reflux symptoms refractory to proton pump inhibitors. Gut 61 (9), 1340–1354. 10.1136/gutjnl-2011-301897 22684483

[B119] Sifrim D. Zerbib F. (2012b). Diagnosis and management of patients with reflux symptoms refractory to proton pump inhibitors. Gut 61 (9), 1340–1354. 10.1136/gutjnl-2011-301897 22684483

[B120] Slaughter J. C. Goutte M. Rymer J. A. Oranu A. C. Schneider J. A. Garrett C. G. (2011). Caution about overinterpretation of symptom indexes in reflux monitoring for refractory gastroesophageal reflux disease. Clin. Gastroenterol. Hepatol. Off. Clin. Pract. J. Am. Gastroenterol. Assoc. 9 (10), 868–874. 10.1016/j.cgh.2011.07.009 21782769

[B121] Souza R. F. Bayeh L. Spechler S. J. Tambar U. K. Bruick R. K. (2017). A new paradigm for GERD pathogenesis. Not acid injury, but cytokine-mediated inflammation driven by HIF-2α: a potential role for targeting HIF-2α to prevent and treat reflux esophagitis. Curr. Opin. Pharmacol. 37, 93–99. 10.1016/j.coph.2017.10.004 29112883 PMC5922421

[B122] Souza R. F. Huo X. Mittal V. Schuler C. M. Carmack S. W. Zhang H. Y. (2009). Gastroesophageal reflux might cause esophagitis through a cytokine-mediated mechanism rather than caustic acid injury. Gastroenterology 137 (5), 1776–1784. 10.1053/j.gastro.2009.07.055 19660463

[B123] Spechler S. J. Hunter J. G. Jones K. M. Lee R. Smith B. R. Mashimo H. (2019). Randomized trial of medical versus surgical treatment for refractory heartburn. N. Engl. J. Med. 381 (16), 1513–1523. 10.1056/NEJMoa1811424 31618539

[B124] Sumi K. Inoue H. Kobayashi Y. Iwaya Y. Abad M. R. A. Fujiyoshi Y. (2021). Endoscopic treatment of proton pump inhibitor-refractory gastroesophageal reflux disease with anti-reflux mucosectomy: experience of 109 cases. Dig. Endosc. Off. J. Jpn. Gastroenterol. Endosc. Soc. 33 (3), 347–354. 10.1111/den.13727 32415898

[B125] Sweis R. Anggiansah A. Wong T. (2010). Efficacy of esophageal impedance/pH monitoring in patients with refractory gastroesophageal reflux disease, on and off therapy. Clin. Gastroenterol. Hepatol. Off. Clin. Pract. J. Am. Gastroenterol. Assoc. 8 (3), 313. 10.1016/j.cgh.2009.09.023 19788931

[B126] Tack J. Pandolfino J. E. (2018). Pathophysiology of gastroesophageal reflux disease. Gastroenterology 154 (2), 277–288. 10.1053/j.gastro.2017.09.047 29037470

[B127] Takeuchi K. Amagase K. (2018). Roles of cyclooxygenase, prostaglandin E2 and EP receptors in mucosal protection and ulcer healing in the gastrointestinal tract. Curr. Pharm. Des. 24 (18), 2002–2011. 10.2174/1381612824666180629111227 29956615

[B128] Talley N. J. Zand I. M. (2021). Optimal management of severe symptomatic gastroesophageal reflux disease. J. Intern Med. 289 (2), 162–178. 10.1111/joim.13148 32691466

[B129] Tanabe T. Hoshino S. Kawami N. Hoshikawa Y. Hanada Y. Takenouchi N. (2019). Efficacy of long-term maintenance therapy with 10-mg vonoprazan for proton pump inhibitor-resistant reflux esophagitis. Esophagus Off. J. Jpn. Esophageal Soc. 16 (4), 377–381. 10.1007/s10388-019-00676-x 31119492

[B130] Toghanian S. Johnson D. A. Stålhammar N. O. Zerbib F. (2011). Burden of gastro-oesophageal reflux disease in patients with persistent and intense symptoms despite proton pump inhibitor therapy: a *post hoc* analysis of the 2007 national health and wellness survey. Clin. Drug Investig. 31 (10), 703–715. 10.2165/11595480-000000000-00000 21756007

[B131] Tominaga K. Iwakiri R. Fujimoto K. Fujiwara Y. Tanaka M. Shimoyama Y. (2012). Rikkunshito improves symptoms in PPI-refractory GERD patients: a prospective, randomized, multicenter trial in Japan. J. Gastroenterol. 47 (3), 284–292. 10.1007/s00535-011-0488-5 22081052

[B132] Tutuian R. Vela M. F. Hill E. G. Mainie I. Agrawal A. Castell D. O. (2008). Characteristics of symptomatic reflux episodes on Acid suppressive therapy. Am. J. Gastroenterol. 103 (5), 1090–1096. 10.1111/j.1572-0241.2008.01791.x 18445095

[B133] Ustaoglu A. Daudali F. A. D’afflitto M. Murtough S. Lee C. Moreno E. (2023). Identification of novel immune cell signature in gastroesophageal reflux disease: altered mucosal mast cells and dendritic cell profile. Front. Immunol. 14, 1282577. 10.3389/fimmu.2023.1282577 38098488 PMC10720318

[B134] Ustaoglu A. Sawada A. Lee C. Lei W. Y. Chen C. L. Hackett R. (2021). Heartburn sensation in nonerosive reflux disease: pattern of superficial sensory nerves expressing TRPV1 and epithelial cells expressing ASIC3 receptors. Am. J. Physiol. Gastrointest. Liver Physiol. 320 (5), G804–G815. 10.1152/ajpgi.00013.2021 33655767

[B135] Ustaoglu A. Woodland P. (2023). Sensory phenotype of the oesophageal mucosa in gastro-oesophageal reflux disease. Int. J. Mol. Sci. 24 (3), 2502. 10.3390/ijms24032502 36768825 PMC9917190

[B136] Vaezi M. F. Fass R. Vakil N. Reasner D. S. Mittleman R. S. Hall M. (2020). IW-3718 reduces heartburn severity in patients with refractory gastroesophageal reflux disease in a randomized trial. Gastroenterology 158 (8), 2093–2103. 10.1053/j.gastro.2020.02.031 32092310

[B137] Vakil N. van Zanten S. V. Kahrilas P. Dent J. Jones R. Global Consensus Group (2006). The Montreal definition and classification of gastroesophageal reflux disease: a global evidence-based consensus. Am. J. Gastroenterol. 101 (8), 1900–1943. 10.1111/j.1572-0241.2006.00630.x 16928254

[B138] Vanuytsel T. Bercik P. Boeckxstaens G. (2023). Understanding neuroimmune interactions in disorders of gut-brain interaction: from functional to immune-mediated disorders. Gut 72 (4), 787–798. 10.1136/gutjnl-2020-320633 36657961 PMC10086308

[B139] Vela M. F. Camacho-Lobato L. Srinivasan R. Tutuian R. Katz P. O. Castell D. O. (2001). Simultaneous intraesophageal impedance and pH measurement of acid and nonacid gastroesophageal reflux: effect of omeprazole. Gastroenterology 120 (7), 1599–1606. 10.1053/gast.2001.24840 11375942

[B140] Vela M. F. Craft B. M. Sharma N. Freeman J. Hazen-Martin D. (2011). Refractory heartburn: comparison of intercellular space diameter in documented GERD vs. functional heartburn. Am. J. Gastroenterol. 106 (5), 844–850. 10.1038/ajg.2010.476 21179012

[B141] Viazis N. Keyoglou A. Kanellopoulos A. K. Karamanolis G. Vlachogiannakos J. Triantafyllou K. (2012). Selective serotonin reuptake inhibitors for the treatment of hypersensitive esophagus: a randomized, double-blind, placebo-controlled study. Am. J. Gastroenterol. 107 (11), 1662–1667. 10.1038/ajg.2011.179 21625270

[B142] Wahlqvist P. Reilly M. C. Barkun A. (2006). Systematic review: the impact of gastro-oesophageal reflux disease on work productivity. Aliment. Pharmacol. Ther. 24 (2), 259–272. 10.1111/j.1365-2036.2006.02996.x 16842452

[B143] Wang H. M. Huang P. Y. Yang S. C. Wu M. K. Tai W. C. Chen C. H. (2023a). Correlation between psychosomatic assessment, heart rate variability, and refractory gerd: a prospective study in patients with acid reflux esophagitis. Life Basel Switz. 13 (9), 1862. 10.3390/life13091862 PMC10533115 37763266

[B144] Wang Y. Lv M. Lin L. Jiang L. (2023b). Randomized controlled trial of anti-reflux mucosectomy versus radiofrequency energy delivery for proton pump inhibitor-refractory gastroesophageal reflux disease. J. Neurogastroenterol. Motil. 29 (3), 306–313. 10.5056/jnm21240 37332140 PMC10334194

[B145] Wang Y. Pan T. Wang Q. Guo Z. (2009). Additional bedtime H2-receptor antagonist for the control of nocturnal gastric acid breakthrough. Cochrane Database Syst. Rev. (4), CD004275. 10.1002/14651858.CD004275.pub3 19821323 PMC12208524

[B146] Woodland P. Aktar R. Mthunzi E. Lee C. Peiris M. Preston S. L. (2015). Distinct afferent innervation patterns within the human proximal and distal esophageal mucosa. Am. J. Physiol. Gastrointest. Liver Physiol. 308 (6), G525–G531. 10.1152/ajpgi.00175.2014 25573174 PMC4360043

[B147] Wu Y. Guo Z. Zhang C. Zhan Y. (2022). Role of the mean nocturnal baseline impedance in identifying evidence against pathologic reflux in patients with refractory gastroesophageal reflux disease symptoms as classified by the Lyon consensus. J. Neurogastroenterol. Motil. 28 (1), 121–130. 10.5056/jnm20277 34980695 PMC8748854

[B148] Xiao Y. Zhang S. Dai N. Fei G. Goh K. L. Chun H. J. (2020). Phase III, randomised, double-blind, multicentre study to evaluate the efficacy and safety of vonoprazan compared with lansoprazole in Asian patients with erosive oesophagitis. Gut 69 (2), 224–230. 10.1136/gutjnl-2019-318365 31409606 PMC6984055

[B149] Xu W. Bai Z. Shang Y. Wang J. Wong Y. Qi X. (2023). Incidence and type of adverse events in patients taking vonoprazan: a systematic review and meta-analysis. Ther. Adv. Gastroenterol. 16, 17562848231167858. 10.1177/17562848231167858 PMC10126681 37113190

[B150] Yadlapati R. Ciolino J. D. Craft J. Roman S. Pandolfino J. E. (2019). Trajectory assessment is useful when day-to-day esophageal acid exposure varies in prolonged wireless pH monitoring. Dis. Esophagus Off. J. Int. Soc. Dis. Esophagus. 32 (3), doy077. 10.1093/dote/doy077 PMC6403452 30124795

[B151] Yadlapati R. Masihi M. Gyawali C. P. Carlson D. A. Kahrilas P. J. Nix B. D. (2021). Ambulatory reflux monitoring guides proton pump inhibitor discontinuation in patients with gastroesophageal reflux symptoms: a clinical trial. Gastroenterology 160 (1), 174–182.e1. 10.1053/j.gastro.2020.09.013 32949568 PMC7755671

[B152] Yamashita H. Kanamori A. Fukuchi T. Tsujimae M. Koizumi A. Iwatsubo T. (2015). Novel prokinetic acotiamide reduces transient lower esophageal sphincter relaxation in healthy subjects. Digestion 92 (2), 90–98. 10.1159/000437301 26279051

[B153] Yamashita H. Okada A. Naora K. Hongoh M. Kinoshita Y. (2019). Adding acotiamide to gastric acid inhibitors is effective for treating refractory symptoms in patients with non-erosive reflux disease. Dig. Dis. Sci. 64 (3), 823–831. 10.1007/s10620-018-5377-9 30465175 PMC6394577

[B154] Yamato M. Nagahama K. Kotani T. Kato S. Takeuchi K. (2005). Biphasic effect of prostaglandin E2 in a rat model of esophagitis mediated by EP1 receptors: relation to pepsin secretion. Digestion 72 (2-3), 109–118. 10.1159/000088365 16172547

[B155] Yang X. Tan J. Liu Y. Feng Y. Shi R. (2022). Comparison of 180° anti-reflux mucosectomy versus 270° anti-reflux mucosectomy for treatment of refractory gastroesophageal reflux disease: a retrospective study. Surg. Endosc. 36 (7), 5002–5010. 10.1007/s00464-021-08857-5 34782965 PMC9160125

[B156] Zdrhova L. Bitnar P. Balihar K. Kolar P. Madle K. Martinek M. (2023). Breathing exercises in gastroesophageal reflux disease: a systematic review. Dysphagia 38 (2), 609–621. 10.1007/s00455-022-10494-6 35842548 PMC9888515

[B157] Zerbib F. Bredenoord A. J. Fass R. Kahrilas P. J. Roman S. Savarino E. (2021a). ESNM/ANMS consensus paper: diagnosis and management of refractory gastro-esophageal reflux disease. Neurogastroenterol. Motil. 33 (4), e14075. 10.1111/nmo.14075 33368919

[B158] Zerbib F. Bredenoord A. J. Fass R. Kahrilas P. J. Roman S. Savarino E. (2021b). ESNM/ANMS consensus paper: diagnosis and management of refractory gastro-esophageal reflux disease. Neurogastroenterol. Motil. 33 (4), e14075. 10.1111/nmo.14075 33368919

[B159] Zerbib F. Roman S. Ropert A. des Varannes S. B. Pouderoux P. Chaput U. (2006). Esophageal pH-impedance monitoring and symptom analysis in GERD: a study in patients off and on therapy. Am. J. Gastroenterol. 101 (9), 1956–1963. 10.1111/j.1572-0241.2006.00711.x 16848801

[B160] Zhang S. Wang X. Xiang X. Yang H. Tang N. Liu L. (2022). A prospective trial to access the optimal circumference of resection in antireflux mucosectomy for treatment-refractory GERD. J. Clin. Gastroenterol. 56 (5), 401–404. 10.1097/MCG.0000000000001650 34974493

